# Central European Woolly Mammoth Population Dynamics: Insights from Late Pleistocene Mitochondrial Genomes

**DOI:** 10.1038/s41598-017-17723-1

**Published:** 2017-12-18

**Authors:** James A. Fellows Yates, Dorothée G. Drucker, Ella Reiter, Simon Heumos, Frido Welker, Susanne C. Münzel, Piotr Wojtal, Martina Lázničková-Galetová, Nicholas J. Conard, Alexander Herbig, Hervé Bocherens, Johannes Krause

**Affiliations:** 1 Institute for Archaeological Science, University of Tübingen, Rümelinstraße 23, 72070 Tübingen, Germany; 2 Department of Archaeogenetics, Max Planck Institute for the Science of Human History, Kahlaische Straße 10, 07745 Jena, Germany; 3 Department of Geosciences, University of Tübingen, Hölderlinstraße 12, 72074 Tübingen, Germany; 4 Senckenberg Centre for Human Evolution and Paleoenvironment (HEP), University of Tübingen, Hölderlinstraße 12, 72074 Tübingen, Germany; 5 Quantitative Biology Centre Tubingen, University of Tübingen, Auf der Morgenstelle 10, 72076 Tübingen, Germany; 6 Department of Human Evolution, Max Planck Institute for Evolutionary Anthropology, Deutscher Pl. 6, 04103 Leipzig, Germany; 7 The Institute of Systematics and Evolution of Animals, Polish Academy of Sciences, Sławkowska 17, 31-016 Kraków, Poland; 8 Department of Anthropology, Faculty of Philosophy and Arts, University of West Bohemia, Sedláčkova 15, 306 14 Pilsen, Czech Republic; 9 Centre for Cultural Anthropology, Moravian Museum, Zelný trh 6, 65937 Brno, Czech Republic; 10 Hrdlicka Museum of Man, Faculty of Science CU, Viničná 7, 128 00 Praha, Czech Republic; 11 Département de Préhistoire du MNHN, Institut de Paléontologie Humaine, 1 rue René Panhard, 75 013 Paris, France; 12 Department of Early Prehistory and Quaternary Ecology, Schloss Hohentubingen, 72070 Tübingen, Germany

## Abstract

The population dynamics of the Pleistocene woolly mammoth (*Mammuthus primigenius*) has been the subject of intensive palaeogenetic research. Although a large number of mitochondrial genomes across Eurasia have been reconstructed, the available data remains geographically sparse and mostly focused on eastern Eurasia. Thus, population dynamics in other regions have not been extensively investigated. Here, we use a multi-method approach utilising proteomic, stable isotope and genetic techniques to identify and generate twenty woolly mammoth mitochondrial genomes, and associated dietary stable isotopic data, from highly fragmentary Late Pleistocene material from central Europe. We begin to address region-specific questions regarding central European woolly mammoth populations, highlighting parallels with a previous replacement event in eastern Eurasia ten thousand years earlier. A high number of shared derived mutations between woolly mammoth mitochondrial clades are identified, questioning previous phylogenetic analysis and thus emphasizing the need for nuclear DNA studies to explicate the increasingly complex genetic history of the woolly mammoth.

## Introduction

Ancient DNA (aDNA) has uncovered complex intra-species population dynamics of the woolly mammoth (*Mammuthus primigenius*). Previously unknown coexistence and extinction events of different maternal lineages have been revealed, changing our understanding of the evolution and extinction of this emblematic megafaunal species. With previous research focusing on short mitochondrial DNA (mtDNA) fragments of the hypervariable region (HVR), two mitochondrial clades across Beringia, clade I (primarily in North America) and clade II (in Siberia), were originally identified^1^. Through similar markers, a third maternal population (clade III) has been recently identified in Europe; a lineage suggested to have been lost around 34 ka cal BP, and replaced through migration of individuals from eastern Eurasia carrying clade I mtDNAs that reached Europe by 32 ka cal BP^2^. Chang *et al* ^3^. reconstructed mitochondrial genomes of clade III individuals from Europe showing that the lineage existed until at least 24 ka cal BP (Dresden, Germany), and possibly until around 12 ka BP in western Eurasia; thus overlapping in time with the arrival of clade I. This is unlike the complete population replacement of mtDNA as suggested by Palkopoulou *et al*.^2^. Furthermore, Enk *et al* ^4^. identified a distinct deeply-diverged sister-lineage of clade III in eastern Eurasia present around 40 cal ka BP (3/B1 in Chang *et al*.^3^). Understanding the nature of replacement events in mammoth mitochondrial lineages has further importance due to previous debate of drift versus selection with the earlier loss of clade II after a period of coexistence with clade I around 44 ka cal BP^1,5,6^. Despite presentation of new dates showing a later survival of clade III in Europe, Chang *et al* ^3^. do not discuss the replacement event in context of the scenario suggested by Palkopoulou *et al*.^2^. Although sampling outside of permafrost environments for aDNA studies has increased, the total number of identified clade I and III individuals in Europe remains limited (*n* = 23 mitochondrial genomes, hypervariable mitochondrial region *n* = 17). Thus, as yet, the turnover event of western Eurasian woolly mammoths have not been extensively explored.

Gaining a greater understanding of mammoth population dynamics across Late Pleistocene Europe also has relevance in smaller geographic regions, due to the unusual interactions of the mammoth with other species at particular archaeological sites. Drucker *et al* ^7^. showed through ^13^C and ^15^N isotopic analysis of bones from the southern German site of Geißenklösterle that the normally distinctive carbon and nitrogen dietary position of the woolly mammoth^8,9^ was atypically being “shared” by horses during both Aurignacian and Gravettian cultural periods (42 ka–~26 ka cal BP^10^); a pattern not found further south-west at Abri Pataud (France). It was hypothesised by the authors^7^ that these mammoths may have belonged to clade III, and thus the stable isotopic pattern of the site may be related to the turnover proposed by Palkopoulou *et al*.^2^.

Here, we aimed to refine understanding of the European late Pleistocene woolly mammoth replacement event in a region that has been sparsely sampled. Furthermore, we do this in the context of unusual isotopic characteristics found at sites in the area. Utilising biomolecular methods (ZooMS, stable isotopes and/or ancient DNA), we screen for mammoth specimens in 48 fragmentary or previously analysed faunal material or collagen samples^7,11–13^, spanning ~38 ka–15 ka cal BP from across central Europe (Supplementary Data S1). Using in-solution enrichment methods on the bone samples, we nearly double the number of dated mammoth mitochondrial genomes from Europe, and in combination with new and previously published stable isotopic and mtDNA data we begin to improve our understanding of late Pleistocene woolly mammoth population turnover in western Eurasia.

## Results

### Collagen Preservation and Stable Isotopes

Collagen of sixteen previously unanalysed specimens from Geißenklösterle and Hohle Fels (Germany) produced here C:N_*coll*_ values of between 2.9–3.6^14^ and had percentages of C_*coll*_ above 8% and N_*coll*_ above 5%^15,16^, representing well preserved and reliable collagen values (Methods and Supplementary Data S2).

The corpus of previously published isotopic data from Geißenklösterle and Hohle Fels from the Late Pleistocene was augmented with the new ^13^C and ^15^N values of mammoth (*n* = 9), rhino (*n* = 6) and horse (*n* = 1) (Supplementary Data S2). Species identification was isotopically inferred through Ward’s minimum variance clustering alongside previously identified specimens (See Methods and Supplementary Information).

The new isotopic values on mammoth were consistent with those measured previously^7,13^, with typical ca. 3–5‰ higher ^15^N values than those of reindeer (Figure S1). The unusual overlap in ^15^N values between horse and mammoth were already described at Geißenklösterle from the same chronological context^7,13^. This unusual pattern at Geißenklösterle is emphasized by the different pattern at Hohle Fels whereby, based on current sampling, horses and mammoths seem to have maintained the ‘isotopic separation’ expected of the classic mammoth steppe during the Aurignacian cultural period (~37 ka cal BP). A single horse found in the Aurignacian-Gravettian transitional layer at Hohle Fels (IIe) suggests that the same pattern may have also occurred at Hohle Fels later - but more stable isotope data of all species from the secure Gravettian layers are required to verify this.

### ZooMS

The osteological remains of woolly mammoth in central Europe, particularly at Geißenklösterle and Hohle Fels, are often highly fragmentary making morphological identification difficult^17^, limiting classification to “mammoth/rhino sized”-like categories^18^. This also often results in variability in DNA preservation. Additionally, previous molecular protocols required large amount of bone material, resulting in large deformations of specimens. In response, curators now often restrict sampling of well preserved morphologically-secure specimens, making access to this material difficult. As ZooMS uses collagen (a biomolecule that generally preserves better than aDNA), this represents a cheap and fast method of confirming species identification when other methods fail^19^.

To improve confidence of uncertain morphological, stable isotopically inferred, and/or DNA-based species assignment (see below) at Geißenklösterle and Hohle Fels (Methods and Supplementary Information), ZooMS was performed on sub-sampled collagen extracted from eleven previously or newly analysed collagen samples for stable isotope analysis (above). Spectral quality was good for all eleven collagen samples analysed, providing direct molecular species identification of the stable isotope results that nine should be identified as Elephantidae and two as Rhinocerotidae (See Supplementary Data S3). For both mammalian families, ZooMS is not able to differentiate between possible Late Pleistocene genera in western Eurasia^20^. For Elephantidae, these specimens probably represent *Mammuthus* (as opposed to *Palaeoloxodon*), and for Rhinocerotidae an attribution to *Coelodonta* seems likely (opposed to *Stephanorhinus*), as per zooarchaeological assessment of the sites^21,22^. ZooMS identifications are in agreement with previous morphological observations as well as dietary isotopic signatures (see Table 1, Supplementary Data S2 and S3).

**Table 1:** Details of late Pleistocene Central European woolly mammoth specimens successfully generating more than three fold coverage and more than 66% complete mitochondrial genomes after insolution enrichment.

Sample	Location	^14^C Date (cal.)	ZooArch Ident.	δ^13^C(‰)	δ^15^N (‰)	ZooMS Ident.	% Endogenous	mtDNA (fold)	mtDNA %	Frag. Length	mtClade
JK2760	Hohle Fels, DE	31812	Mammoth/Rhino Size	−21.0	8.5	—	4.296	86.54	98	80.95	III
JK2761	Hohle Fels, DE	—	Mammoth/Rhino Size	−21.3	8.3	—	0.222	5.29	72	79.02	III
JK2762	Geißenklösterle, DE	37904	Mammoth/Rhino Size	−21.0	9.0	Elephantidae	0.215	22.23	93	79.04	III
JK2764	Geißenklösterle, DE	38031	Mammoth	−21.1*	8.0*	Elephantidae	0.606	243.18	98	73.35	III
JK2765	Hohle Fels, DE	32226*	Mammoth/Rhino Size	−21.1	8.5	Elephantidae	0.902	109.99	97	70.40	III
JK2766	Hohle Fels, DE	35127*	Mammoth	−21.1*	8.0*	—	1.019	79.49	97	79.00	III
JK2768	Hohle Fels, DE	31666	Mammoth/Rhino Size	−20.8	8.5	Elephantidae	1.72	60.40	98	87.13	III
JK2769	Geißenklösterle, DE	37360	Mammoth	−21.1*	8.9*	Elephantidae	0.342	27.95	93	74.68	III
JK2770	Hohle Fels, DE	31683	Mammoth/RhinoSize	−20.8	8.4	—	2.728	164.57	98	82.25	III
JK2771	Geißenklösterle, DE	—	Mammoth	−21.2*	8.8*	—	0.126	8.89	82	82.26	III
JK2772	Hohle Fels, DE	38336	Mammoth/Rhino Size	−21.1	9.0	—	6.018	230.59	98	73.93	III
JK2773	Hohle Fels, DE	34924	Mammoth/Rhino Size	−21.1	9.0	Elephantidae	0.429	29.41	95	79.81	III
JK2774	Geißenklösterle, DE	—	Mammoth	−21.1*	9.1*	—	0.256	5.68	74	74.37	III
JK2779	Geißenklösterle, DE	—	Mammoth	−20.9*	8.3*	—	0.038	11.63	86	78.58	III
JK2780	Geißenklösterle, DE	31762*	Mammoth	−20.9*	8.7*	Elephantidae	0.048	7.27	77	78.24	III
JK2782	Kesslerloch, CH	15409*	Mammoth	−20.5*	6.4*	—	22.694	108.30	99	90.14	I
JK2790	Kraków Spadzista, PL	26966*	Mammoth	−20.3*	8.7*	—	0.765	216.37	98	72.02	I
JK2796	Kraków Spadzista, PL	22961*	Mammoth	−20.3*	9.0*	—	0.453	6.72	78	67.91	I
JK2802	Kraków Spadzista, PL	—	Mammoth^†^	−20.8*	9.5*	—	15.563	522.97	99	77.40	I
JK2803	Kraków Spadzista, PL	26947*	Mammoth	−20.2*	9.0*	—	0.091	11.86	85	68.59	I

Radiocarbon (^1^4C) dates were calibrated with OxCal 4.2^62^ to the Intcal 13 curve^63^. Endogenous DNA calculation is derived from shotgun sequencing of the woolly mammoth NGS libraries. Fragment lengths are in base pairs (bp). Asterixis (*) indicate previously published data. Dagger (†) represents individuals with tooth furrows. More detailed summaries can be found in Supplementary Data S1–S6.

### Ancient DNA

For ancient DNA analysis, 44 bone and teeth samples were selected from Kesslerloch (Switzerland), Kraków Spadzista (Poland), Předmostί (Czech Republic), and additional individuals from Geißenklösterle and Hohle Fels to those analysed above. A summary of analysis performed per sample can be found in Supplementary Data S1.

In-solution capture of mammoth mitochondrial genomes was successfully carried out with capture baits generated by long-range PCR using three pairs of newly designed primers (Supplementary Data S4) on DNA extracted from elephant blood (see Methods and Supplementary Information). Of the forty-four samples genetically tested, after capture twenty produced mitochondrial genomes meeting the ‘relaxed’ criteria of Chang *et al* ^3^. (see Table 1 and methods), resulting in 5.3 to 522.97-fold coverage with 72%–99% of the mtDNA genome covered (Table 1). Additionally, of these, 13 met the ‘strict’ Chang *et al* ^3^. criteria of >10-fold average coverage with 90% nucleotide support (range x 22.2–x522.97) and >80% (range 81%–98%) of the mitochondrial genome covered. Capture efficiency (endogenous DNA percentage after/before capture) of these 20 mtDNAs ranged from 0.8%–78.9% (Supplementary Data S5). All mitochondrial genomes had C to T damage patterns indicative of authentic aDNA^23^ (Supplementary Figure S3). The number of samples for each site producing mitochondrial genomes is as follows: Kesslerloch (*n* = 1), Geißenklösterle (*n* = 7), Hohle Fels (*n* = 8), Kraków Spadzista (*n* = 4). Shotgun data from the Předmostί specimens all presented low values for endogenous DNA (0.005%–0.024%, Supplementary Data S5), suggesting bad DNA preservation at the site. Both Kraków Spadzista and Předmostί are open air rather than the cave environments of the other sites, the former type making specimens more susceptible to weathering, temperature fluctuation, pH and precipitation changes, which could lead to variable levels of DNA preservation^24^.

To confirm isotopic observations and ZooMS taxonomic identification, all shotgun libraries were mapped to a white rhinoceros assembly (*Ceratotherium simum simum*; NCBI Accession: GCA_000283155.1). A ten-fold increase in DNA mapping to the rhinoceros genome sequence versus a hybrid elephant/mammoth genome sequence (see Methods) identified the morphologically identified “mammoth/rhino size” Hohle Fels rib fragment sample HF_67565_89_1632_Va as rhinoceros (Supplementary Data S5), in agreement with isotopic and ZooMS observations. When sufficient DNA preservation was present, all other “mammoth/rhino size” samples from Geißenklösterle and Hohle Fels taxonomically identified through isotopes or ZooMS were confirmed as mammoth.

The mitochondrial genomes from Geißenklösterle, Hohle Fels, Kraków Spadzista and Kesslerloch were aligned to those analysed in Chang *et al* ^3^. with *Elephas maximus* (NCBI Accession: EF588275) as outgroup, for a total of one hundred and sixty-four mitochondrial sequences spanning Eurasia and North America. The addition of the mtDNAs reconstructed here increases the number of European mitochondrial genomes (*n* = 22 in Chang *et al* ^3^. to *n* = 42). Due to inconsistent gaps in the alignment caused by different reconstruction methods across previous publications, the D-Loop (positions 15,422–16,770 on the ‘Krause’ reference sequence) was removed for downstream analysis.

Of the twenty samples preserving enough DNA for complete mitochondrial genome reconstruction, the Geißenklösterle and Hohle Fels specimens consisted of only clade III, whereas Kraków Spadzista and Kesslerloch only contained clade I individuals - representing the earliest mitochondrial genomes of the migrating clade I population in central Europe.

**Figure 1:**
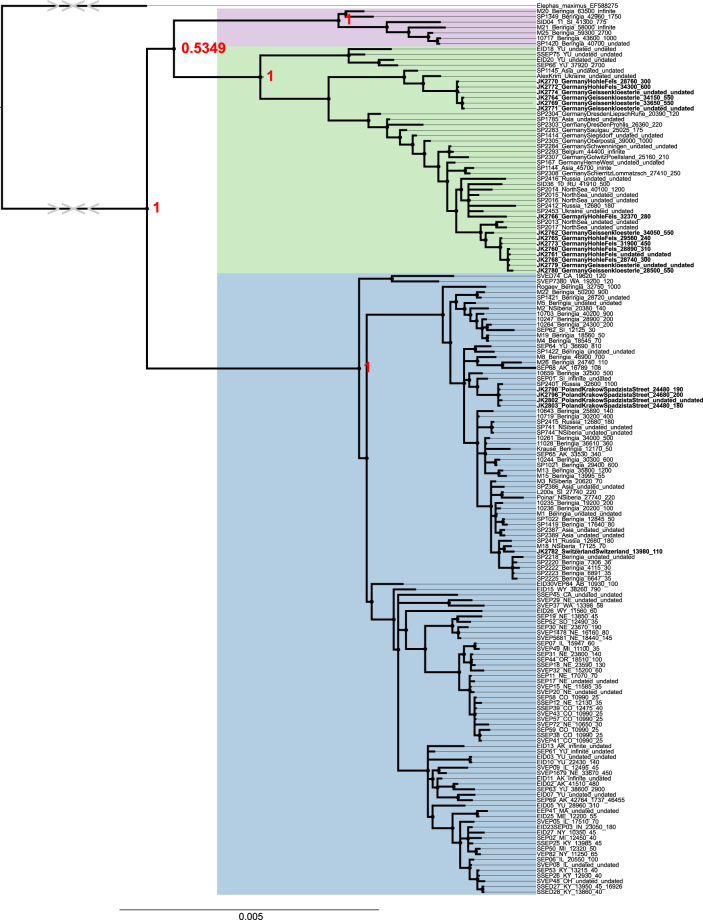
Bayesian phylogenetic tree of all previously published woolly mammoth mammoths mitochondrial genomes^3–5,25–27^ and 20 mitochondrial genomes presented here (in bold). Node values represent posterior probability values from 100,000,000 steps for the bayesian tree. For visibility reasons, only support values related to the relationship of the three clades are shown. Clades are indicated by the following colours: Blue - I; Purple - II; Green - III. Radiocarbon dates in sample names are uncalibrated. The full tree as well as Neighbour-Joining, Maximum Parsimony and Likelihood trees can be found in Supplementary Figures S4–S7. Trees were generated using Figtree (tree.bio.ed.ac.uk/software/figtree/) and modified in Inkscape (inkscape.org).

Clustering of the samples into three clades agrees with previous mammoth mitochondrial analyses^1–5,25^. However it was observed that here, as well as in all studies presenting Bayesian or Maximum Likelihood (ML) phylogenetic trees including all three mtDNA clades, the node connecting clade II and III in the majority of trees had low posterior probability or bootstrap values (Fig. 1) - regardless of phylogenetic method, evolutionary model and sequence completeness. While the BEAST coalescence tree from Enk *et al*.^4^ showed stronger support on this node (in contrast to ML), the Chang *et al*.^3^ tree that includes many more clade III mitochondrial sequences again shows low support. This low support could be considered to represent a trifurcation in the relationship between the three clades as explicitly presented in the ML tree presented by Enk *et al*.^4^, thus making these relationships difficult to resolve. This was further explored with our dataset by generating Neighbour-Joining (NJ), Maximum Parsimony (MP), ML, and Bayesian trees (see Methods and Supplementary Information) which again showed a low statistical support on the node connecting clade II and III (Fig. 1, Supplementary Figures S3–S6). As this suggested that there are potential difficulties in the ability to resolve the relationships between the three clades, an additional test to assess sequence relationships between each possible pairing of clades was performed by comparing the number of ‘shared’ derived SNPs between each clade (see Supplementary Information). This identified a rather high number of ‘homoplasic’ sites - the same mutations occurring independently on each clade, rather than following the expected phylogenetic relationships - suggesting that a typical ‘strict’ tree-like evolution in woolly mammoth mitochondrial lineages may not have occurred. The MP tree identified 65 derived positions for clade I, 70 for clade II, and 50 for clade III. In contrast, the average number of ‘shared’ derived SNPs sites are as follows: between clade II and clade I 24, between clade II and III 27, between clade I and III 17; out of approximately 15.4 thousand base pairs. The majority of the protein-coding gene related SNPs were found to be synonymous (*n* = 25), and the non-synonymous positions (*n* = 4) were distributed across the entire genome with no clustering on particular genes (Supplementary Data S6, Supplementary Information).

## Discussion

A variety of methods have been used for the reconstruction of woolly mammoth mitochondrial genomes, including multiplex PCR^3,5,26,27^, shotgun sequencing^5,25,28^, array capture^3^, and in-solution capture with *in silico* designed baits^4^. Here, the in-solution capture method of Maricic *et al* ^29^. was to our knowledge for the first time successfully applied to mammoth specimens, utilising DNA baits amplified from elephant blood using only three pairs of primers. Baits generated in-house from DNA of related species is likely to be more cost-effective than commercial kits (as used in^4^) as well as expressing less reference bias for individual mtDNA lineages while producing similarly high mtDNA average coverages. We have nearly doubled the number of radiocarbon dated European clade III genomes (>3-fold coverage, *n* = 12 and *n* = 11 respectively). Five out of six tested “mammoth/rhino” sized bones actually belong to mammoths, which increases the proportion of securely identified mammoth remains found in the archaeological assemblages at Geißenklösterle and Hohle Fels. The identification of rhinoceros specimens as a byproduct of our main analysis highlights the benefits of screening otherwise species-of-interest ‘negative’ aDNA libraries or collagen extracts from ‘undetermined’ bone. Screening aDNA reads of off-target samples by mapping to different genomes (as above) requires no extra laboratory work, and collagen fingerprinting methods such as ZooMS can be cheaply applied to leftovers of collagen extraction for isotopic analysis or radiocarbon dating^30,31^. For example, ZooMS was still able to confirm the zooarchaeological identification for the Geißenklösterle sample JK2778 (GK_11499_58_279_IIb) when genetic identification failed.

Analysis of woolly mammoth mitochondrial genomes performed here has shown that the low statistical support for the relationship between clade I, II and III can be potentially attributed to a similarly high number of ‘shared’ derived SNPs between each pair of clades; complicating the resolving of the maternal phylogenetic tree. These derived SNPs comprise of around one third or more of the derived positions present on each branch between the MRCA of all clades and MRCA of each clade. To ensure these positions are real, the mapping strategy used in this study removes potential NUMTs fragments (nuclear-mitochondrial sequences) that could cause false SNP calls (despite the low likelihood of this happening^25^) by removing any fragment that mapped to both nuclear and mitochondrial genomes. We also find these homoplasic positions remain even when comparing mitochondrial genomes produced by different methods (multiplex PCR, array capture, in-solution capture and shotgun sequencing), suggesting this is not a protocol artefact. Additionally, the consensus genomes generated here were called with a penalising filter for damage, making typical aDNA damage, increased frequency of C to T miscoding lesions at the end of fragments, also unlikely to be the cause of these ‘homoplasies’. This was confirmed by visual inspection of read alignments. Biologically, although not the only possibilities, this pattern is consistent with processes such as recombination or convergent evolution. This would be unusual as mitochondria are typically considered unlikely to undergo recombination as having more than one mitochondrial haplotype after fertilisation is rare^32,33^, and convergent evolution is expected to generally lead to a higher number of non-synonymous than synonymous mutations^33^. Although not an issue for the aims of this study, this pattern should be further scrutinised as this could suggest current interpretations of the maternal evolutionary history of the mammoth may be more complicated than previously thought. As the statistical support and number of ‘shared’ derived SNPs seem to show that the data does not follow a tree-like pattern, it may be that it is not possible to apply dating or classical tree-based phylogenetic methods on mammoth mitochondrial data^34^. This could possibly call into question the reliability of the Bayesian dating analyses performed previously^2–5^.

**Figure 2:**
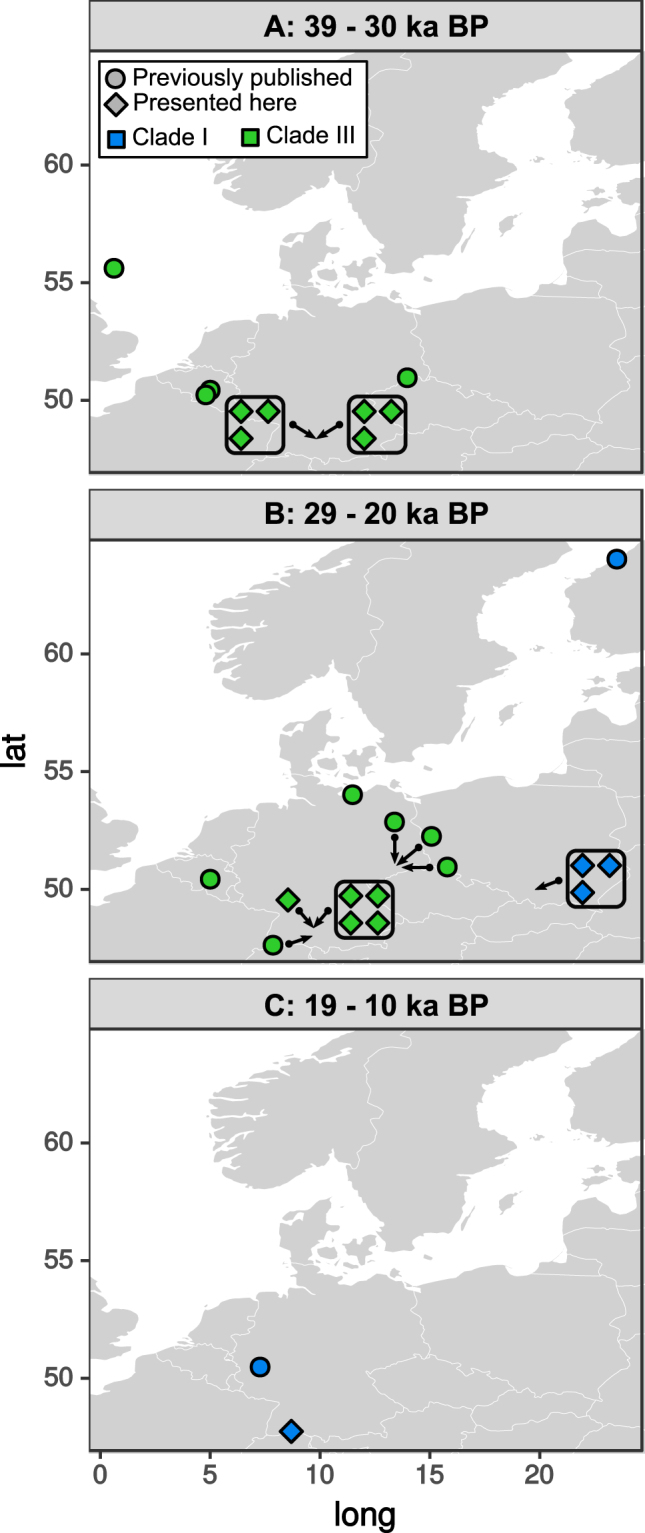
Time series of all genetically typed and radiocarbon dated European woolly mammoths from this study (diamonds), and dated HVR samples and full mitochondrial genomes analysed in^2,3^ (circles). Time slices are in uncalibrated BP. Clade III lineages (green) are present until 25 ky BP in Germany, but clade I (blue) is now shown to be present in central Europe by 24 ka BP (27 ka cal BP), as represented by the new Kraków Spadzista mitochondrial sequences. Maps were generated in R^60^ using the ggplot2 package^61^ and modified in Inkscape (inkscape.org).

New radiocarbon dates of the identified mammoth samples presented here (Table 1, Supplementary Data S1) confirm the continuation of the existence of clade III mtDNA^3^ after 34 ka cal BP in Europe. Whilst there is no mixture of individuals from the two clades at the sites analysed in this study, these data do not yet reject the possibility of a coexistence being found in eastern Europe between clade III and clade I. Indeed, the Kraków Spadzista mitochondrial genomes indicate that clade I was already established in central Europe by 27 ka cal BP, much further west and earlier than previously detected^2,3^ (Fig. 2). Thus, the increased sampling of Europe (Chang *et al*.^3^ this study) is indicating that our understanding of this replacement event may be less clear than ‘just’ a extirpation of clade III and subsequent arrival of clade I with no ‘temporal overlap’ as proposed previously^2^. Indeed, a synthesis of all mammoth dates in Europe by Lorenzen *et al* ^35^. and Puzachenko *et al* ^36^. has shown mammoth remains being present in Europe throughout this period.

The increasing number of mtDNA sequences from western Eurasia is uncovering a curiously similar pattern to one that occurred ten thousand years earlier in eastern Eurasia between clade I and clade II. Barnes *et al* ^1^. originally concluded that drift was the likely cause of the loss of clade II in a restricted population. In contrast, further Bayesian skyline analysis by Debruyne *et al* ^6^. did not detect a demographic bottleneck that would result from genetic drift in clade II, and thus put forward the possibility of some form of competitive replacement causing the rather sudden loss, when considered in the context of the timing of the arrival of clade I just prior to the loss of clade II. However, Gilbert *et al* ^5^. at the same time suggested that the mitochondria themselves did not show any functional differences that could provide some form of selective advantage. Both arguments could also potentially be applied to the situation occurring in Europe between clade III and I ten thousand years later. More recent BEAST analysis on whole Eurasian datasets by Palkopoulou *et al* ^2^. did not detect any noticeable reduction of the effective population size during this period. Nuclear DNA would potentially allow to test for functional differences between all three populations, defined by mtDNA clades, in particular why clade I seems to be more ‘dominant’ than the other two clades. This type of data would be particularly pertinent due to the ‘contradictions’ identified in our study in the homoplasies observed between the different clades along the mitochondrial genome, thus questioning our ability of current models to elucidate the intra-population dynamics of the woolly mammoth.

Generated from highly fragmentary remains, the 20 central European Late Pleistocene mitochondrial genomes produced here increases the number of mitochondrial genome sequences from this region. Unusual patterns and difficulties in resolving the relationship between the three clades due to sequences not seemingly following a strict ‘binary’ tree-like pattern have been raised here. This questions previous demographic and genetic dating analyses and thus requires further investigation and scrutiny of these ancient data. The new mitochondrial genomes represent the earliest European clade I individuals at 27 ka cal BP, and corroborate the existence of clade III in Europe past 34 ka cal BP. Increasing numbers of samples are reducing the ‘temporal gap’ between the respective occupations of the two clades in Europe. This pattern is starting to indicate parallels with a replacement event that occurred previously between clade I and clade II in Beringia, however the nature of both events remain unresolved and are becoming even less clear than previously considered. More genetic sampling from chronologically secured sites in Europe before and throughout the LGM is required to confirm a loss of clade III before the loss of clade I. Analysis of male lineage markers as well as autosomal chromosomes is required to help disentangle the increasingly complex relationship of different mammoth groups and may provide additional evidence for the local and global extinction of one of the largest members of the Pleistocene megafauna.

## Methods

### Sample Selection and Zooarchaeological Identification

Forty-four samples spanning ~34 ka to 13 ka BP (38 ka −15 ka cal BP) were obtained from archaeological collections or previous stable isotopic analysis^7,11–13^ from the Late Pleistocene sites: Kesslerloch (Switzerland, *n* = 1), Geißenklösterle (Germany, *n* = 12), Hohle Fels (Germany, *n* = 10), Předmostί (Czech Republic *n* = 6), and Kraków Spadzista (Poland, *n* = 15) (See Supplementary Data S1). Three further collagen samples from Geißenklösterle that were previously radiocarbon dated^10^ were analysed for stable isotopic and ZooMS analysis. An additional collagen sample from Hohle Fels from previous dating was also analysed for carbon and nitrogen stable isotopes.

For samples from Geißenklösterle and Hohle Fels, a zooarchaeological identification of woolly mammoth was not always possible due to high fragmentation of remains (especially of ribs, an important raw material for the production of tools at the site^13^). Although the megafaunal remains also include woolly rhino, identifiable remains from this species are not as abundant; therefore the megafaunal remains are considered to be dominated by woolly mammoth in the Aurignacian and Gravettian cultural layers^22^. In cases of unclear identification the size class of “mammoth/rhino” was assigned (Supplementary Data S1).

The samples from all sites were additionally chosen based on previously published direct ^14^C dates or secure stratigraphic layers prior the Last Glacial Maximum ranging from ~36 ka–~19 ka ^14^C (based on dates of archaeological layers) and a single individual after the Last Glacial Maximum dated to 13 ka BP (Kesslerloch). Overall, the samples selected here represent the date range of the estimated extinction of clade III and earliest European clade I woolly mammoths^2,3^.

Bone fragments from Geißenklösterle and Hohle Fels were sampled from ribs, vertebrae, a skull and a radius. From Kraków Spadzista, the dentine of the molars and the bone from a mandible were taken using a dremel circular saw. The remaining samples came from previous isotopic analysis in the form of bone or tooth powder, or collagen^11–13^.

### Collagen Extraction and Isotopic Analysis

For collagen preservation screening, bone powder remaining from aDNA sampling (see below) from Geißenklösterle and Hohle Fels was analysed through elemental analysis^37^, performed on a Vario EL III elemental analyzer (Department of Geography, University of Tübingen). Collagen was extracted following a modified Longin^38^ protocol as by Bocherens *et al*.^39^. Collagen δ^13^C and δ^15^N values were then measured on a ThermoQuest Delta + XL mass spectrometer (Department of Geosciences, University of Tübingen). International standards for ^13^C (V-PDB) and ^15^N (atmospheric air) were used and measurements were normalised to δ^13^C values of USGS24 (δ^13^C = −16.00) and to δ^15^N values of IAEA 305 A (δ^15^ *n* = 39.80). Analytical error, based on within-run replicate measurements of laboratory standards (albumen, modern collagen, USGS 24, IAEA 305 A) was ±0.1 for δ^13^C and ±0.2 for δ^15^N.

Collagen from the isotopic analysis of specimens yielding mitochondrial genomes (see below) and that had not been previously dated was additionally sub-sampled for radiocarbon dating at the Oxford Radiocarbon Accelerator Unit (ORAU). ^14^C dates were converted to F ^14^C dates and then calibrated in OxCal 4.2 using the IntCal13 calibration curve. Calibrated ^14^C dates are indicated by ‘cal BP’ (Supplementary Data S1).

Cluster analysis was performed in JMP v11.1.1 (SAS) using Ward’s minimum variance.

### ZooMS Identification

Collagen extracted from samples from Geißenklösterle and Hohle Fels during isotopic analysis was subsampled (<1 mg dry collagen) for collagen fingerprinting by Zooarchaeology by Mass Spectrometry (ZooMS^40^). Eleven samples either identified as “mammoth” or “rhino” were chosen to provide within-site spectral references when assessing samples assigned “mammoth/rhino size” from morphological and isotopic observations. These included seven samples from Geißenklösterle and four from Hohle Fels (Supplementary Data S2). ZooMS MALDI-TOF-MS analysis was conducted following Welker *et al*.^41^, (see Supplementary Information) and peptide markers were identified against the ZooMS sequence database presented by Welker *et al*.^20^. ZooMS relies on collagen type I (COL1) amino acid sequence variation to differentiate between closely related species. As collagen contains less substitutions compared to genetic sequences and ZooMS only uses a subset of the phylogenetic information present in COL1^42^, ZooMS taxonomic identifications are commonly on the (sub)family level. Hence, for Elephantidae standard ZooMS screening is not able to differentiate between *Mammuthus* and *Palaeoloxodon*, the two Late Pleistocene Elephantid genera present in Eurasia^43^.

### Sample Preparation, DNA Extraction and Sequencing

All aDNA work prior to amplification was performed in a dedicated aDNA laboratory at the University of Tübingen, following aDNA clean room protocols^44^. In brief, the positive pressure laboratory is UV irradiated daily and surfaces regularly cleaned with bleach, and separate dedicated hoods for reagent preparation, sampling and reactions are used. All laboratory consumables and reagents are UV irradiated when possible. Full clean room suits with hairnets, masks and double pairs of gloves are worn. Positive extraction controls in the form of an internal well-characterised cave bear bone were included, and one negative control during each extraction and two controls per library preparation session.

aDNA extraction was performed using a modified version of a silica-based protocol^45^. Extracts were converted to double indexed sequencing libraries^46,47^ and transferred to a modern DNA lab for amplification (Supplementary Data S4).

Enrichment of mitochondrial DNA was performed following the protocol of Maricic *et al*.^29^. Template material was provided by Wilhelma Zoo (Stuttgart, Germany) as a subsample of blood taken from two Asian elephants after routine veterinary care by the in-house Veterinary practitioner. Blood was drawn using standard venipuncture techniques in accordance with the relevant veterinary regulations and practises of the Zoo. No experimentation was performed on the elephants for the purpose of this study. The DNA extracted using a QIAamp DNA Blood Mini kit (Qiagen). Three new primer pairs (Supplementary Data S4) were manually designed to produce three ~5.5 k bp amplicons spanning the entire *Elephas maximus* mitochondrial genome (NCBI Accession: EF588275.2) through Long-Range PCR (LR-PCR). Amplicons sonicated to an length of <1000 bp were converted to biotinylated bait libraries and in-solution bead capture was performed^29^.

Pre- and post-enrichment libraries were then sequenced on an Illumina HiSeq 2500 (University of Tübingen). More information about methods from all laboratory protocols can be found in the Supplementary Information.

### aDNA Pre-Processing, Consensus Calling and Phylogenetic Analysis

Endogenous DNA quantification, aDNA authentication and mitochondrial genome reconstruction were performed with an in-house version of the NGS data processing pipeline EAGER v1.92^48^ using a hybrid reference consisting of the African elephant nuclear genome and the woolly mammoth mitochondrial genome (NCBI Accession: GCA_000001905.1 and NC_007596.2 respectively).

The final consensus sequences were called by GenConS (Generate Consensus Sequence, v1), a new consensus caller tool designed here to reduce the risk of false positive SNP positions that may be erroneously called due to typical aDNA damaged molecules^49,50^. In brief, taking a SNP and optionally an InDel VCF file as input (both were used in this study), a position is analysed to assess whether an aDNA miscoding pattern^49^ is present prior to SNP calling. If a damage pattern is detected, the coverage of the miscoding base is artificially reduced in order to reduce the chance of a false-positive SNP call due to damage. Additional thresholds apply: a minimum coverage for a position to be called (5 in this study) and a minimal allele frequency for a call to made (75% in this study). When either are not met an ‘N’ is called. The resulting consensus sequence is written in FASTA format. Furthermore, a file in Consensus Call Format is generated giving detailed information of each consensus call (further information about GenConS can be found in the Supplementary Information). GenConS is available in the TOPAS package at www.github.com/subwaystation/TOPAS.

Previously published mitochondrial genomes^3–5,25–27,51^ were downloaded from the NCBI SRA database, and the consensus sequences generated here were aligned with these using ‘Consensus Align’ in Geneious vR8^52^. Evolutionary models were estimated using modelgenerator v85^53^, jModelTest2 v2.1.10^54^ and IQ-Tree v1.4.2^55^, and phylogenetic analysis performed using Geneious vR8 for Neighbour Joining, MEGA for Maximum Parsimony^56^, IQ-Tree v1.4.2 for Maximum Likelihood^57,58^, and MrBayes v3.2.6 for Bayesian^59^ methods (Supplementary Information, Supplementary Data S6).

### Accession Codes

Sequencing libraries have been deposited in the European Nucleotide Archive (ENA) with the project accession PRJEB21582. Final mitochondrial sequences have been stored in the NCBI GenBank database with accession numbers MF579931-MF579950.

## Electronic supplementary material

Supplementary Information

Supplementary Dataset S1

Supplementary Dataset S2

Supplementary Dataset S3

Supplementary Dataset S4

Supplementary Dataset S5

Supplementary Dataset S6
