# Supplementary material for: Central European Woolly Mammoth Population Dynamics: Insights from Late Pleistocene Mitochondrial Genomes
Source: Sci Rep. 2017 Dec 18;7:17714. doi: 10.1038/s41598-017-17723-1 (PMC5735091; doi:10.1038/s41598-017-17723-1)
Supplement: Supplementary file 1 — Supplementary Information [file 41598_2017_17723_MOESM1_ESM.pdf]

Central European Woolly Mammoth Population Dynamics: Insights from Late Pleistocene Mitochondrial Genomes - Supplementary Information

James A. Fellows Yates[1,2,*], Dorothée G. Drucker[3,4], Ella Reiter[1], Simon Heumos[1,5], Frido Welker[6], Susanne C. Münzel[1], Piotr Wojtal[7], Martina Lázničková-Galetová[8,9,10,11], Nicholas J. Conard[4,12], Alexander Herbig[1,2], Hervé Bocherens[3,4], and Johannes Krause[1,2,]**

[1]Institute for Archaeological Science, University of Tübingen, Rümelinstraße 23, 72070 Tübingen, Germany
[2]Department of Archaeogenetics, Max Planck Institute for the Science of Human History, Kahlaische Straße 10, 07745, Jena, Germany
[3]Department of Geosciences, University of Tübingen, Hölderlinstraße 12, 72074 Tübingen, Germany
[4]Senckenberg Centre for Human Evolution and Paleoenvironment (HEP), University of Tübingen, Hölderlinstraße 12, 72074 Tübingen, Germany
[5]Quantitative Biology Centre Tübingen, University of Tübingen, Auf der Morgenstelle 10, 72076 Tübingen, Germany
[6]Department of Human Evolution, Max Planck Institute for Evolutionary Anthropology, Deutscher Pl. 6, 04103 Leipzig, Germany
[7]The Institute of Systematics and Evolution of Animals, Polish Academy of Sciences, Sławkowska 17, 31-016 Kraków, Poland
[8]Department of Anthropology, Faculty of Philosophy and Arts, University of West Bohemia,Sedláčkova 15, 306 14 Pilsen, Czech Republic
[9]Centre for Cultural Anthropology, Moravian Museum, Zelný trh 6, 65937 Brno, Czech Republic
[10]Hrdlicka Museum of Man, Faculty of Science CU, Viničná 7, 128 00 Praha, Czech Republic
[11]Département de Préhistoire du MNHN, Institut de Paléontologie Humaine, 1 rue René Panhard, 75 013 Paris, France
[12]Department of Early Prehistory and Quaternary Ecology, Schloss Hohentübingen, 72070 Tübingen, Germany
[*]krause@shh.mpg.de

Supplementary Note: Archaeological Contexts

Kesslerloch

Kesslerloch is a cave site situated at 447m above sea level near the Swiss-German border in the Kanton of Schaffhausen (Switzerland)[1]. The site was originally discovered in 1873-4 by Konrad Merk[2], excavated by Jakob Nüesch and Jakob Heierli in the early 20th century[3] and most recently excavated by Marcel Joos and Jürg Sedlmeier in 1980[1].

AMS radiocarbon dating of the occupational layers have yielded dates placing the site during the Magdalenian cultural period, ranging from approximately ~14k BP to 10k BP[1]. These include dates of a suggested early 'dog' at ~12.2k BP[4] and a woolly mammoth from around ~13.9k BP[5]; matching the archaeological artefacts associated with the Magdalenian[3]

Pollen analysis showed that the majority of the layers reflect 'Oldest-Dryas' conditions, including low amounts of tree pollen in contrast to higher amounts in post-glacial layers and higher amounts of shrubland plants[6], reflecting a tundra like environment[1].

The faunal assemblage from the site contains a range of mammoth-steppe related species[2]. Although dominated by reindeer[1,3] and horse[1,2], it also contains other species including carnivores such as wolves and wolverines[7], as well as the suggested early domesticated dog[4]. Smaller species include snow hare and some evidence of birds including ptarmigan.

A range of Magdalenian flint[6], bone artefacts has been found, including bone needles and tooth amulets[2], as well as antler artefacts including a reindeer engraved on an reindeer antler[3]. Evidence of wood burning (including a hearth[1]) as well as an assemblage of edible plants that are not expected to naturally congregate in the cave were found at the site[6].

Geißenklösterle

Geißenklösterle Cave stands at an elevation of 585 m asl about 60 m above the Ach Valley near the town of Blaubeuren, south west Germany. Shortly after the discovery of the site Gustav Riek conducted a small test excavation. In 1973 Eberhard Wagner excavated Geißenklösterle following an episode of illegal digging. The site is best known for Joachim Hahn's long-term excavations between 1974 and 1991, which led to the discovery of rich deposits from the Gravettian and the Aurignacian and more modest results from the Magdalenian and the Middle Palaeolithic. Excavations by Nicholas Conard in 2001 and 2002 examined the basal Aurignacian deposits and extended the sequence through thick Middle Palaeolithic layers to bedrock.

The stratigraphic sequence at Geißenklösterle includes five Middle Palaeolithic horizons (VIII-IV), two main Aurignacian horizons (III and II), as well as Gravettian, Magdalenian and Mesolithic strata belonging to various subunits of archaeological horizon I[8].

Geißenklösterle is well known for its Aurignacian finds including four small sculptures carved from mammoth ivory, and three flutes - two carved from bird bones and one from mammoth ivory[9–12]. The Aurignacian begins between 42 ky and 41 ky cal. BP, as has been confirmed by a wide range of radiocarbon, luminescence and U-series dates[8,13–15]. This early date suggests an early migration of modern humans up the Danube Valley[14] and the finds of figurative art and musical instruments count among the oldest found.

Mammoths were the largest and most imposing animals on the steppes of Ice Age Eurasia and play a prominent role in the archaeology of the Swabian Jura and of Geißenklösterle. While isotopic studies have suggested that Neanderthal ate large amounts of meat and marrow from mammoth, the zooarchaeological record from the caves of the Swabian Jura indicate that horses, reindeer and ibex counted among the key prey during both the Middle and Upper Palaeolithic[16]. Fish, small mammals and birds play a more significant role in the diet of all phases of the Upper Paleolithic than during the Middle Palaeolithic[17]. Remains of mammoth are only rarely associated with Middle Palaeolithic deposits but are common in the Aurignacian and Gravettian find horizons. During the Aurignacian mammoth ivory served as a key raw material for numerous tools, as well as for artworks, musical instruments and personal ornaments. People of the Gravettian frequently carved ornaments from ivory and developed a range of new tools made from mammoth bones, particularly ribs[18]. After the Last Glacial Maximum mammoths played only very modest role in the economies of the Magdalenian, but depictions of mammoths are well-known in various Magdalenian contexts across Europe, but not in the Swabian Jura.

Hohle Fels

Hohle Fels Cave is situated 534 m asl and 7 m above the Ach Valley floor, close to the town of Schelklingen and ca. 2 km from Geißenklösterle cave. The cave is a large hall of 500 m^2 with a height of 30 m.

The discovery of Hohle Fels goes back to the 19th century. After extraction of clay for pottery and bat's guano as fertilizer, large quantities of cave bear bones were recognized. This caught the interest of pastor Theodor Hartmann, who informed Oscar Fraas, curator of the 'Königliche Naturalienkabinet' in Stuttgart. In 1870/71 Hartmann and Fraas conducted an excavation digging out most of the huge hall[19]. All finds were transported to Stuttgart, where most of them were destroyed during World War II. The first excavation after the war was a sondage in the entrance tunnel conducted by Gertrud Matschak and Gustav Riek, which is not documented. From 1977 to 1979 and 1987 to 1996 Joachim Hahn lead excavations in the same area in Hohle Fels. After Hahn's death research was continued by Nicholas Conard, and the excavations continue until today.

The stratigraphic sequence at Hohle Fels includes four Middle Palaeolithic layers (IX-VI), covered by several horizons from the Aurignacian (Vb-IId) and followed by Gravettian layers (IIc and IIb). Finally, an occupation during the Magdalenian is documented (IIa-I)[20,21].

Simone Riehl and colleagues used charcoal, pollen and phytolith analysis to reconstruct the environment of the Aurignacian to Magdalenian[22]. They identified a cold, pine-dominated tundra during the Aurignacian and Gravettian (with increasingly colder temperatures during the Aurignacian) but with warmer and wetter willow-dominated environments during the Aurignacian-Gravettian transition[22]. Despite low preservation quality of the organic material, this corroborated earlier micromorphological work of a cold Gravettian by Paul Goldberg et al.[21], and the whole sequence by Chris Miller at Geißenklösterle and Hohle Fels[23]. However, this contradicted previous multi-site research across the whole of Europe suggesting slight warming during the end of the Aurignacian[24].

Hohle Fels is best known for its Aurignacian finds including four mammoth ivory figurines, a horse head, a water fowl, a 'little lion man', and a Venus figurine[14,25,26]. Other important finds are a flute from a vulture radius, and fragments of other bird and ivory flutes[26]. The dating of the Aurignacian is very much the same as for the Geißenklösterle (see above).

The faunal record for all cave sites in the Ach Valley is that of a mammoth-steppe, including megafaunal species such as mammoth, woolly rhino, and horse, reindeer, but also cervids like red deer, sometimes roe deer and giant deer[16]. In all caves cave bear is a dominant species, hibernating and giving birth to cubs, and a lithic projectile point sticking in a thoracic vertebrae of a cave bear has been found[16,27]. Traces of butchering on cave bear bones were already known from Geißenklösterle[28], but Hohle Fels provides the first proof of its hunting. Together with foetal horse bones and infantile mammoth calves (also found in

the other Ach Valley caves), the faunal remains points to a human hunting season of horses in winter and mammoth in spring[16].

Předmostí

Předmostí (Moravia, Czech Republic) is a spatially extensive complex of open-air sites, situated in a strategic location in the southern mouth of the Moravian Gate (Chlum hill – 17.270 longitude, 49. 272 latitude). These sites was first excavated in 1880-1882, however they were initially exploited rather than systematically explored. Systematic excavations in recent years have been directed by Bohuslav Klíma (1971-1973, 1975-1976, 1982-1983) and Jiří Svoboda (1989-1992, 2002, 2008). The latter excavations have produced radiocarbon dates from between 26 ky and 24 ky BP[29]. Subsequent calibrated dates range from 34 ky to 29 ky cal BP[30]

Předmostí Ia–b and III is a standard Upper Palaeolithic – Gravettian site[29]. The Předmostí complex has yielded faunal remains[31], lithics[32], bone tools and weapons, works of art and human personal ornaments[33,34]. The locality was inhabited all year round by humans[35].

The Gravettian occupation layer in Předmostí contained charcoal of fir, hazel, oak and hornbeam[36]. According to the results of analysis of Gravettian soil samples[37], the pollen spectrum is poor in identified species and enriched in Fagus. The dry, steppe-like character is indicated by the presence of *Poaceae*, *Chenopodiaceae* and *Botrychuium* pollen, and *Urtica* indicates anthropogenic activities. Human settlement activity at Předmostí falls within the very end of a moderate period, when the climate gradually began to cool down and ended in an extremely cold glacial maximum (LGM)[38].

The faunal assemblage of Předmostí (for first time published by Pokorný[31]) contains mammoth (more than 1,000 individuals), followed by wolf and fox, reindeer, hare and wolverine[38]. The spectrum of game found at Předmostí I is dominated by mammoth (72.05 %)[38] and other megafauna, represented by rhinoceros and megaloceros. Mammoth bones are found in accumulations where skeletal parts are preserved in anatomical order and some bones are sorted by type. Classification of mammoths from Předmostí on the basis of metrical and morphological characteristics is presented in the publication by Robert Musil[39] and pathological changes by Alina Krzemińska *et al.*[40].

Předmostí I has been classified as an Upper Palaeolithic burial site[41] on the basis of an assemblage of human skeletons redefined as early modern humans[42]. According to a reconstruction of the burial site by Klíma[43], which was carried out on the basis of a diary by Maška – discoverer of this assemblage - mammoth bones were found in the vicinity of human remains. Some belonged to the enclosure of this area, with some mammoth scapulae likely used as a protective cover for human skeletons – identified on the basis of later analogous graves from Dolní Věstonice 3 and Pavlov 1[29]. Humans inhabiting Předmostí I hunted mammoths[38] and used them as a source of food[44], material (bones, tusks) for construction elements of dwellings[45], household tools, weapons, works of art and personal ornaments[33,34,46].

Kraków Spadzista

Kraków Spadzista is located on the high northern headland of Blessed Bronisława Hill (Kraków, Poland). It is situated at a height connected with the main summit of the hill. The east and west contains a Pleistocene depression but the north side consists of a rocky cliff. Currently the site is located in the area of a 19th century Austrian fortification. The site was accidentally discovered in late autumn 1967 and excavations that started in 1968 continue up to today, in total covering eleven trenches. Both archeological and paleontological material were discovered in all trenches. Most of the finds were discovered in layer 6; a tundra gley which had developed in a soliflucted clay loam deposited under a moist and harsh climate[47,48]. It should be noted that some archaeological artefacts were collected from older (layer 7) and younger (layer 5) sediments[49,50]. A series of radiocarbon dates suggests that the site was occupied from about 17 ky to 25 ky uncal BP (~22 ky to 29 ky cal BP)[51,52].

Kraków Spadzista is one of the best known Gravettian sites in Europe. The cultural level is well visible in all trenches. It includes characteristic tools and among others shouldered points and Kostienki knives[53]. Fieldwork performed over the last few decades at Kraków Spadzista has yielded more than 26,000 mammal remains[53]. The osteological material is dominated by woolly mammoth, which belongs to a minimum 97 individuals[51]. Other Pleistocene mammals species are represented by isolated finds of single individuals. One exception is Arctic fox, the remains of which were found at a larger amount (NISP=2428; MNI=30)[54]. The most impressive feature of the site is a huge accumulation of mammoth bones near the rocky cliff. In a very small area (trenches B+B1), about 150 sqm., the remains of a minimum of 86 woolly mammoth individuals were discovered[49,51]. More than six thousand specimens (NISP=6375) from all parts of the mammoth skeleton (cranial and postcranial) have been identified. In addition, 16,000 fragmentary osteological remains have been collected. The dimensions and thickness of cortical part of the fragments suggest that these most likely come from fragmented mammoth bones[51].

In total, ten trenches have been excavated at Kraków Spadzista, localized in an area of about a few hundred square meters. Kraków Spadzista is interpreted as one large site with different zones of Gravettian hunter-gatherer activity[51]. Zone I, which covers the camp area, includes trenches C, C2, C3, and D. Zone II, consisting of workshop and animal-processing areas, include trenches E, E1 and F. Trenches B+B1, B "workshop", III and V lie in the zone III – a mammoth bone accumulation (a dump area). Gravettian hunters probably killed mammoth at the zone III or close to it, as suggested by traseological and taphonomic data[49,51,53].

Supplementary Methods: Isotopic Analysis

For each specimen, a small fragment was carefully sawn with a rotary tool equipped with a circular diamond-coated blade, then ultrasonicated in acetone and water, rinsed with distilled water, dried and crushed to a powder of 0.7 mm grain size[55]. Then, an aliquot of around 5 milligrams was used to measure the nitrogen content (%N) of the whole bone, in order to screen out samples with excessive collagen loss[56]. For instance, fresh bones contain 4% nitrogen while ancient bones with less than 0.4% nitrogen usually fail to yield good collagen[56]. The measurements were performed using a Vario EL III elemental analyzer using Sulfanilic acid from Merck as internal standard. The mean standard errors were better than 0.05% for %N.

The collagen was purified according to a well-established protocol[55]. The elemental and isotopic measurements were performed at the Isotopic Geochemistry unit of the Department of Geosciences at the University of Tübingen (Germany), using an elemental analyzer NC 2500 connected to a Thermo Quest Delta+XL mass spectrometer. The elemental ratios C:N were calculated as atomic ratios (Supplementary Data S2). The isotopic ratios are expressed using the "δ" (delta) value as follows:

$$\delta^{13}C = \frac{(^{13}C/^{12}C)_{sample}}{(^{13}C/^{12}C)_{reference} - 1}\, x1000‰ \quad (1)$$

$$\delta^{15}N = \frac{(^{15}N/^{14}N)_{sample}}{(^{15}N/^{14}N)_{reference} - 1}\, x1000‰ \quad (2)$$

The international references are V-PDB for δ^{13}C values and atmospheric nitrogen (AIR) for δ^{15}N values. Measurements were normalized to δ^{13}C values of USGS24 (δ^{13}C = -16.00‰) and to δ^{15}N values of IAEA 305A (δ^{15}N = 39.80‰). The reproducibility was ±0.1‰ for δ^{13}C measurements and ±0.2‰ for δ^{15}N measurements, based on multiple analyses of purified collagen from modern bones.

The reliability of the isotopic signatures of the extracted collagen was addressed using their chemical composition (%C, %N, and C/N ratio). These values must be similar to those of collagen extracted from fresh bone to be considered reliable for isotopic measurements and radiocarbon dating. Several studies have shown that collagen with atomic C/N ratios lower than 2.9 or higher than 3.6 is altered or contaminated, and should be discarded, as well as extracts with %N < 5%[57].

The new isotopic data corroborates the pattern previously seen at Geißenklösterle by Drucker *et al.*[58] (Supplementary Figure S1). Additionally, cluster analysis was applied on the 27 samples of mammoth, rhino and mammoth/rhino size using Ward's minimum variance method performed on the stable carbon and nitrogen isotopic composition using the JMP software (SAS, version 11.1.1). The ^{13}C and ^{15}N values were organised in two main groups. One of them can be divided into two subgroups - cluster 1 in green, cluster 2 in blue - both being nearer to each other than from cluster 3 in red (Supplementary Figure S2). Cluster 1 and 2 differed in their ^{13}C values, while exhibiting comparable ^{15}N values. Cluster 3 showed no overlap with cluster 1 and 2 in both ^{13}C and ^{15}N values. All the samples that could be attributed to rhino based on ZooMS and/or aDNA evidence, if not on morphology, were assigned to cluster 3. All the specimens that could be assigned to mammoth species, following on one or more of the different approaches, were found in cluster 1 and 2. We thus consider that based on stable isotopic values the mammoth/rhino size samples from cluster 3 (P-22973) and from cluster 1 or 2 (JK2761, JK2768) as belonging to rhino and mammoth, respectively.

Supplementary Methods: ZooMS Analysis

Eleven dried collagen samples from Hohle Fels and Geißenklösterle extracted for isotopic analysis (see above) were subsampled (<1 mg) for ZooMS analysis. Dried collagen was resuspended in ammonium-bicarbonate buffer (50 mM) and denatured at 65°C for 1 hour[59]. Trypsin digestion, C18 ZipTip purification and peptide elution followed Welker *et al.*[60]. MALDI-TOF-MS spectra were acquired in the mass range 900-4000 m/z, with taxonomic identifications based on an extended ZooMS peptide marker library presented elsewhere[61]. Full sample details and ZooMS taxonomic results can be found in Supplementary Data S3.

Supplementary Methods: Ancient DNA Analysis

Extraction and Library preparation

For bone fragment samples both sides were placed under UV light for 30 minutes, and a dentist drill was used to remove a thin layer of the bone surface that was then discarded. Powder from previous analyses were not placed under UV. For all samples approximately 50µg of powder was drilled, or directly measured (in the case of samples from previous analyses) and DNA extracted following the protocol by Dabney *et al.*[62], eluting in 100µl of TET. Extracts were converted into double indexed,

double stranded libraries following the protocol of Meyer and Kircher[63,64] using 20µl of extract. Each library (including extraction and library controls) were quantified using 1/1 and 1/100 dilutions with IS7 and IS8 qPCR primers[63] on a Lightcycler 96 system (Roche) with the DyNAmo Flash SYBR Green qPCR kit (Biozym). The average library contained a total of 3.62E+10 copies, whereas negative controls 2.29E+06 copies (Supplementary Data S4, low numbers of possible contaminating reads from controls was also confirmed through mapping to the Elephant genome - Supplementary Data S5). Each library was then indexed using two unique indices after splitting the library based on the concentration values calculated from qPCR using a Pfu-Turbo reaction kit (Agilent)[64], purified by MinElute column (Qiagen) and eluting in 50µl TET. All steps prior indexing PCR were performed in a dedicated clean room at the University of Tübingen, following established aDNA anti-contamination protocols. Indexed libraries were then also quantified by qPCR as above with primers IS5 and IS6[63] in a physically separate laboratory, to confirm the successful indexing of each library (Supplementary Data S4). Libraries were then amplified following Scheunemann *et al.*[65], using an AccuPrime Pfx kit (Invitrogen). In the event of low amplification efficiency, the amplification was repeated with the same parameters on the products of the previous amplifications. Amplified libraries were purified again by MinElute column and quantified with an Agilent 2100 Bioanalyzer DNA 1000 Chip (Supplementary Data S4).

Mitochondrial Capture and Sequencing

DNA from elephant blood drawn using standard venipuncture techniques during routine veterinary care was subsampled and stored in PAXgene Blood DNA (Qiagen) and Vacuette EDTA (Greiner Bio-One) tubes and frozen at -20oC. DNA was extracted from the blood using an QIAamp DNA Blood Mini kit (Qiagen), following the manufacturer's instructions alongside negative controls. Three sets of primers were manually designed to span the entire *Elephas maximus* mitochondrial genome (NCBI Accession No.: EF588275.2) as reference, and checked for species specificity by using BLAST[66] to the NCBI Nucleotide database (Supplementary Data S4).

Long-range PCR was then performed using the elephant DNA extracts and an Expand Long Range dNTPack kit (Roche), following manufacturer's instructions using cycler parameters in Supplementary Data S4. An aliquot of the amplification products were checked for expected lengths on 1% agarose gel, and then the products purified with MinElute column following manufacturer's instructions, except eluted in 130µl of low TE buffer (Applichem). The purified amplicons were then sheared to below one kilobase using Covaris a microTUBE.

Following Maricic *et al.*[67], sheared elephant DNA was converted to baits and used for enrichment of mammoth mitochondrial DNA in equimolar pools of 5 libraries each. Enriched libraries were then purified with MinElute columns and eluted in 15µl TET. Libraries were then quantified using qPCR as before, and then amplified using a Herculase II Fusion PCR kit (Agilent) to 1.00E+13 copies. Post-capture amplification products were purified with MinElute columns as per manufacturer's instructions and quantified with an Agilent 2100 Bioanalyzer DNA chip, as before. Pre- and Post- Libraries and blanks were then sequenced separately on an Illumina HiSeq 2500, with a 150bp paired end sequencing kit, yielding around 1 million reads for each library for both pre- and post-enriched libraries.

Data Preprocessing, aDNA authentication and consensus calling

Sequencing reads for all libraries were de-multiplexed and de-indexed using bcl2fastq (V1.8.4, Illumina). The EAGER v1.92.10 pipeline[68] was then used on default settings (unless stated otherwise) to process the resulting fastq files.

For the unenriched 'shotgun' libraries, preprocessing consisted of FASTQC[69] for assessing sequencing quality and ClipAndMerge[68] was used to merge paired reads and remove adaptors. BWA (-l 32, -n 0.01[70]) was used for mapping reads to 1) a hybrid of the African elephant nuclear genome and the woolly mammoth mitochondrial genome (NCBI Accession: GCA_000001905.1 and NC_007596.2 respectively) and 2) white rhino (*Ceratotherium simum simum*) assembly (NCBI Accession: GCA_000283155.1), without any mapping quality filter. Duplicates were removed with DeDup[68], mapping quality assess with Qualimap[71] and aDNA authentication was performed with mapDamage[72] (Supplementary Figure S3).

For the mitochondrially enriched libraries, EAGER was ran with 'mtCapture' mode, which allows the removal of NUMTs by mapping all reads to the entire genome, but only performing downstream analysis on mitochondrial reads. The pipeline was run as above, however reads were mapped with CircularMapper[68] (-l 32, -n 0.01) and with a mapping quality filter of 37. Additionally, GATK UnifiedGenotyper[73–75] was used for SNP and InDel calling.

To reduce the number of false SNP calls in low coverage genomes due to damaged ends of reads[76], a new consensus caller tool Generate Consensus Sequence (GenConS, available in the TOPAS package at: www./github.com/subwaystation/TOPAS) was developed. GenConS reads a FASTA reference and a single corresponding GATK Unified Genotyper VCF file of a single sample of which a consensus sequence is created. Before calling a consensus base it is analysed whether the position has a C at the reference and a T as a variant, or a G at the reference and an A as a variant, determining if an aDNA miscoding pattern is present or not. In the first case, the coverage of the miscoding base (T or A) is multiplied by a punishment ratio of default value 0.8 in order to account for possible aDNA damage. In the latter case, the number of reads of each base are left as given. As a result, the consensus base is the base with the highest coverage at the current position fulfilling the following (default) criteria: a) the base is covered by at least five reads, and b) the fraction of the reads containing the base is at least 75%. If

any of these requirements are not satisfied, an N is added to the consensus sequence. All declared ratios and filters are the current standard values implemented in the tool and can be configured by the user. If available, an additional VCF containing INDELs can be included into the call evaluation process. The incorporation of INDELs into the consensus sequence occurs as follows: An insertion is treated like a non-damaged SNP and extends the consensus sequence. However, when a situation occurs where some reads show a deletion and others not, the information of the deletion itself (originating from the INDELs file) and the information of the (possible) SNPs that are shown by the reads that do not have the deletion are taken into account. The coverage from the reads containing the deletion (unpunished) is then weighed up against the coverage of the corresponding SNPs. If the SNPs win, each SNP position is evaluated as mentioned before. If the deletion wins, the consensus sequence is shortened according to the deletion. Having no 'winner' at all, all deletion spanning positions become 'N'. The resulting consensus sequence is written in FASTA format. Furthermore, the tool produces a consensus call file (CCF) giving detailed information of each consensus call.

Consensus sequences were generated for each sample by applying GenConS to the VCF files produced from EAGER, following the 'Relaxed' and 'Strict' criteria of Chang *et al.*[77], requiring a position to be covered by at least 3 reads, of which the SNP called must be in least of the 3 reads and C to Ts penalised with a score of 0.8. The woolly mammoth mitochondrial genome used in mapping was used as a reference for consensus calling.

Alignment and Phylogenetic Analysis

Sequences that reached the 'Relaxed' criteria of Chang *et al.*[77] of at least 3 fold coverage and having at least 66% of the genome complete (including *Elephas maximus* NCBI Accession No: EF588275), were aligned along with the all genomes used by Chang *et al.*[77] using ConsensusAlign on default settings in Geneious vR8[78]. Due to differences in genome reconstruction methods of all previously published mitochondrial genomes, the entire D-loop (as annotated on the 'Krause reference sequence, NCBI Accession: NC_007596.2) was removed.

Evolutionary substitution models of the entire mitochondrial sequence were estimated using ModelGenerator v0.85[79], IQ-Tree v1.4.2[80] and jModelTest v2.1[81] allowing 4 gamma categories. The former two gave the TN+G model[82] as most suitable with the BIC criteria - as found previously by Gilbert *et al.*[83] and was subsequently used here, whereas jModelTest2 gave the HKY+G model. Models with invariant sites were not consisted due to the intraspecific nature of the data[84,85].

Geneious vR8 was used to generate a Neighbour-Joining tree, with 1000 bootstraps and the TN model. MEGA v6[86] was used to generate a Maximum Parsimony tree with 1000 bootstraps, using all sites and with the Subtree-Pruning-Regrafting search method. IQ-Tree 1.4.2[80] was used to generate a Maximum Likelihood tree, also with 1000 bootstraps and the TN+G4 model.

MrBayes v3.2.6[87] was used to generate a Bayesian phylogenetic tree. An approximation of the TN+G model was set with the following parameters: nst=6 and rates=gamma. The analysis was run for 100,000,000 steps sampling every 10,000. Average ESS of posterior probabilities were greater than 100 (>156, Supplementary Data S6) , and trace plots visually verified for stability.

In all cases *Elephas maximus* was used as an outgroup for rooting the phylogenetic tree. All four trees can be seen in Fig. 2 and Supplementary Figures S4 - S7.

Analysis of 'Shared' Derived SNPs

All trees from previous mitochondrial analyses[77,84,88] have the same low statistical support on the clade connecting node despite using different data (short fragments versus complete mitochondrial genomes), models (partitioned vs. non-partitioned), and methods (Bayesian vs. ML). Despite using full mitochondrial genomes (considered to be more robust than single genes[89]), and using a non-partitioned model (unlike Chang *et al.*[77]), Bayesian trees produced here in MrBayes show the same unresolved topology for the three major mammoth mitochondrial clades. This was explored further by running a suite of phylogenetic methods (Neighbor Joining - NJ, Maximum Parsimony - MP, Maximum Likelihood - ML) which resulted in two possible topologies: clade II diverging from clade I after diverging from clade III (NJ), and clade II diverging from clade III after divergence from clade I (MP, ML, Bayesian), the latter matching previous publications[77,84] Although bootstrapping and posterior probabilities are not directly comparable, in all cases low statistical support was found on the node representing the divergence of the three clades. This is independent of the inclusion (previous publications) or exclusion (this publication) of the D-Loop regions. This is further reflected in the short branch between the most recent common ancestor of all three clades and that of clade II and III in the MP tree, with 18 positions defining this branch.

To investigate this further, nine samples were selected (three from each clade) where genome reconstruction had been performed with different methods (where possible) and all with average coverages of at least x10 to account for biases in mitogenome reconstruction methods and for potential false-positive SNP calls due to damage. The following samples were selected: Clade I - SP1022, JK2802, Krause; Clade II - M20, M21, 10717; Clade III - JK2764, SP1144, SP312. Alignments of every possible three-way comparison (i.e. one individual from each clade compared to a Elephas maximus reference

sequence, NCBI Accession: EF588275) were extracted from an alignment performed on all these sequences using Geneious ConsensusAlign. As an initial test for assessing the sequence relationships between all three clades, a custom R (v3.2.2)[90] script utilising the 'Ape' package[91] was written to count the number of derived positions that were shared between clades in a pairwise comparison between individuals of each combination of clade to the Elephas maximus outgroup (Supplementary Figure S8). The number of derived SNPs shared across each possible grouping (e.g. clade I sharing a derived position with clade II, with clade III retaining the reference position; clade I sharing with clade III and II with the reference etc.) was then averaged (Supplementary Data S6). Additionally, the counts of positions from each combination were concatenated to include single entries for each position (Supplementary Data S6). A schematic for this calculation can be seen in Supplementary Figure S8. For SNP effect analysis, to ensure these bases were related to the relevant woolly mammoth positions rather than *Elephas maximus*, every possible 'homoplasic' positions were converted back to the positions on the 'Krause' reference sequence by cross-referencing the positions on the original alignment (Supplementary Data S6). Therefore only those homoplasic positions found specifically in clade II or clade III were considered. These positions were then processed by SnpEff v3.1[92] compared to a custom-built annotation database based on the woolly mammoth 'Krause' genome (NCBI Accession: NC_007596.2), including a 100bp upstream-downstream allowance (Supplementary Data S6)

References

1. Napierala, H. *Die Tierknochen aus dem Kesslerloch Neubearbeitung der paläolithischen Fauna*, vol. 2 of *Beiträge zur Schaffhauser Archäologie* (Baudepartement des Kantons Schaffhausen, Kantonsarchäologie, Schaffhauen, 2008).
2. Merk, C. *Excavations at the Kesslerloch Near Thayngen Switzerland: A Cave of the Reindeer Period* (Longmans, Green and Co., London, 1876).
3. Weinstock, J. *Late Pleistocene Reindeer Populations in Middle and Western Europe: An Osteometrical Study of Rangifer Tarandus*, vol. 3 of *BioArchaeologica* (Mo Vince Verlag, Tübingen, 2000).
4. Napierala, H. & Uerpmann, H.-P. A 'new' palaeolithic dog from central Europe. *Int. J. Osteoarchaeol.* **22**, 127–137 (2012).
5. Stuart, A. J., Sulerzhitsky, L. D., Orlova, L. A., Kuzmin, Y. V. & Lister, A. M. The latest woolly mammoths (*Mammuthus primigenius* Blumenbach) in Europe and Asia: a review of the current evidence. *Quat. Sci. Rev.* **21**, 1559–1569 (2002).
6. Ammann, B. *et al. Neue Untersuchungen am Kesslerloch bei Thayngen/SH: Sondierbohrungen im östlichen Vorplatzbereich und ihre naturwissenschaftlich-achäologische Auswertung*, vol. 17 of *Antiqua* (Verlag Schweizerische Gesellschaft für Ur- und Frühgeschichte, Basel, 1988).
7. Bocherens, H. *et al.* Isotopic evidence for dietary ecology of cave lion (*Panthera spelaea*) in north-western Europe: Prey choice, competition and implications for extinction. *Quat. Int.* **245**, 249–261 (2011).
8. Higham, T. *et al.* Testing models for the beginnings of the Aurignacian and the advent of figurative art and music: the radiocarbon chronology of Geißenklösterle. *J. Hum. Evol.* **62**, 664–676 (2012).
9. Hahn, J. *Kraft und Aggression. Die Botschaft der Eiszeitkunst im Aurignacien Süddeutschlands?*, vol. 7 of *Archaeologica Venatoria* (Tübingen Verlag, Tübingen, 1986).
10. Hahn, J. *Die Geißenklösterle-Höhle im Actal bei Blaubeuren I. Fundhorizontbildung und Besiedlung im Mittlepaläolithikum und im Aurignacien* (Theiss, Stuttgart, 1988).
11. Hahn, J. & Münzel, S. Knochenflöten aus dem Aurignacien des Geißenklösterle bei Blaubeuren, Alb-Donau-Kreise. *Fundberichte aus Baden-Württemberg* **20**, 1–12 (1995).
12. Conard, N. J., Malina, M., Münzel, S. C. & Seeberger, F. Eine Mammutelfenbeinflöte aus dem Aurignacien des Geissenklösterle: Neue Belege für eine musikalische Tradition im Frühen Jungpaläolithikum auf der Schwäbischen Alb. *Archäologisches Korrespondenzblatt* **34**, 447–462 (2004).
13. Richter, D., Waiblinger, J., Rink, W. J. & Wagner, G. A. Thermoluminescence, electron spin resonance and ^{14}C-dating of the late Middle and early Upper Palaeolithic site of Geißenklösterle Cave in southern Germany. *J. Archaeol. Sci.* **27**, 71–89 (2000).
14. Conard, N. J. & Bolus, M. Radiocarbon dating the appearance of modern humans and timing of cultural innovations in Europe: new results and new challenges. *J. Hum. Evol.* **44**, 331–371 (2003).
15. Richard, M. *Chronologie des occupations humaines au Pléistocène supérieur dans le Jura Souabe, Allemagne et dans les vallées de la Saône et du Rhône, France, par les méthodes de la résonance de spin électronique et des séries de l'uranium, ESR/U-Th.* Ph.D. thesis, National Museum of Natural History, Paris. (2015).

16. Münzel, S. C. & Conard, N. J. Change and continuity in subsistence during the Middle and Upper Palaeolithic in the Ach Valley of Swabia (south-west Germany). *Int. J. Osteoarchaeol.* **14**, 225–243 (2004).

17. Conard, N. J., Kitagawa, K., Krönneck, P., Böhme, M. & Münzel, S. C. The importance of fish, fowl and small mammals in the Paleolithic diet of the Swabian Jura, southwestern Germany. In *Zooarchaeology and Modern Human Origins*, Vertebrate Paleobiology and Paleoanthropology, 173–190 (Springer Netherlands, 2013).

18. Barth, M. M., Conard, N. J. & Münzel, S. C. Palaeolithic subsistence and organic technology in the Swabian Jura. In Fontana, L., F.-X., C. & Bridault, A. (eds.) *In Search of Total Animal Exploitation. Case Studies from the Upper Palaeolithic and Mesolithic:*, vol. 2040 of *BAR International Series 2040*, 5–20 (John and Erica Hedges, Oxford, 2009).

19. Fraas, O. Beiträge zur culturgeschichte aus schwäbischen höhlen entnommen. der hohle fels im achthal. *Archiv für Anthropologie* **5**, 173–213 (1872).

20. Conard, N. J. & Bolus, M. Radiocarbon dating the late middle paleolithic and the aurignacian of the swabian jura. *Journal of Human Evolution* **55**, 886–897 (2008).

21. Goldberg, P., Schiegl, S., Meligne, K., Dayton, C. & Conard, N. J. Micromorphology and site formation at hohle fels cave, swabian jura, germany. *Eiszeitalter und Gegenwart* **53**, 1–25 (2003).

22. Riehl, S., Marinova, E., Deckers, K., Malina, M. & Conard, N. J. Plant use and local vegetation patterns during the second half of the late pleistocene in southwestern germany. *Archaeological and Anthropological Sciences* 1–17 (2014).

23. Miller, C. *Formation processes, palaeoenvironments, and settlement dynamics at the Palaeolithic cave sites of Hohle Fels and Geißenklösterle: a geoarchaeological and micromorphological perspective*. Ph.D. thesis, Eberhard-Karls-Universität Tübingen (2009).

24. Huijzer, B. & Vandenberghe, J. Climatic reconstruction of the weichselian pleniglacial in northwestern and central europe. *Journal of Quaternary Science* **13**, 391–417 (1998).

25. Conard, N. J. De nouvelles sculpture en ivoire aurignaciennes du jura souabe et la naissance de l'art figuratif. neue elfenbeinskulpturen aus dem aurignacien der schwäbischen alb und die entstehung der figürlichen kunst. In Floss, H. & Rouquerol, N. (eds.) *Les chemins de l'art Aurignacien en Europe. Colloque International, Aurignac, 16-18 Septembre 2005*, 317–330 (Éditions Musée-forum, Aurignac, France, 2007).

26. Conard, N. J., Malina, M. & Münzel, S. C. New flutes document the earliest musical tradition in southwestern germany. *Nature* **460**, 737–740 (2009).

27. Münzel, S. C. Seasonal hunting of mammoth in the Ach-Valley of the swabian jura. In Caverretta, G., Gioia, P., Muss, M. & Palombo, M. R. (eds.) *The World of Elephants - Proceedings Of The First International Congress, Rome 2001*, 318–322 (Consiglio Nazionale delle Ricerche - Roma, Rome, 2001).

28. Münzel, S. C. Seasonal activities of human and non-human inhabitants of the geissenklösterle-cave, near blaubeuren, Alb-Danube district: Old world hunters and gatherers. *Anthropozoologica* 355–361 (1997).

29. Svoboda, J. The upper Paleolithic burial area at Předmostí: ritual and taphonomy. *J. Hum. Evol.* **54**, 15–33 (2008).

30. Musil, R. Palaeoenvironment at Gravettian sites in central Europe with emphasis on Moravia (Czech Republic). *Quartär* **57**, 95–123 (2010).

31. Pokorný, M. Příspěvek k paleontologii diluvia v Předmostí u Přerova. *Časopis Moravského musea zemského* **XXXVI**, 33–52 (1951).

32. Oliva, M. Pavlovienská sídliště u Předmostí. *Acta Musei Moraviae, Sciences sociales* **LXXXII**, 3–64 (1997).

33. Valoch, K. Ornamentale gravierungen und ziergegenstände von Předmostí bei Přerov in Mähren. *Anthropologie* **XIII**, 81–91 (1975).

34. Valoch, K. Die beingeräte von Předmostí bei Přerov in Mähren (Tschechoslowakei). *Anthropologie* **XX**, 57–69 (1982).

35. Nývltová Fišáková, M. Seasonality of Gravettian sites in the middle Danube Region and adjoining areas of central Europe. *Quat. Int.* **294**, 120–134 (2013).

36. Musil, R. The Middle and Upper Palaeolithic game suite in central and southeastern Europe. In van Andel, T. H. & Davies, W. (eds.) *Neanderthals and modern humans in the European landscape during the last glaciation: Archaeological results of the Stage 3 Project*, Short Run Press, 167–190 (ambridge, McDonald Institute for Archaeological Research, Cambridge, 2003).

37. Svoboda, J., ložek, V., Svobodová, H. & Škrdla, P. Předmostí after 110 years. *J. Field Archaeol.* **21**, 457–472 (1994).

38. Musil, R. The paleoclimatic and paleoenvironmental conditions at Předmostí. In Velemínská, J. & Brůžek, J. (eds.) *Early Modern Humans from Předmostí. A new reading of old documentation*, 15–20 (Academia Prague, Prague, 2008).

39. Musil, R. Morfologická a metrická charakteristika předmosteckých mamutů. *Actes Musei Moraviae* **XLIII**, 95–107 (1958).

40. Krzemińska, A., Wojtal, P. & Oliva, M. Pathological changes on woolly mammoth (*Mammuthus primigenius*) bones: Holes, hollows and other minor changes in the spinous processes of vertebrae. *Quat. Int.* **359–360**, 178–185 (2015).

41. Ullrich H. Předmostí - an alternative model interpreting burial sites. *Anthropologie* **34**, 299–306 (1996).

42. Veleminska, J. & Brůžek., J. *Early Modern Humans from Predmosti near Prerov, Czech Republic: A new reading of old documentation* (Prague Academia, Prague, 2008).

43. Klíma, B. Das Paläolithische massengrab von Předmosti, versuch einer rekonstruktion. *Quartär, 41/42 (1991)* 187–194 : Abb. (1991).

44. Bocherens, H. *et al.* Reconstruction of the Gravettian food-web at Předmostí I using multi-isotopic tracking (^{13}C, ^{15}N, ^{34}S) of bone collagen. *Quat. Int.* **359–360**, 211–228 (2015).

45. Valoch, K. Contribution to the architecture of Upper Palaeolithic dwellings. *Anthropologie* **XXV**, 115–116 (1987).

46. Lázničková-Galetová, M. La gravure sur l'ivoire de mammouth en Moravie - les cas de Dolni Věstonice I et Předmostí I (République Tchèque). In Cleyet-Merle, J.-J., Geneste, J.-M. & Man-Estier, E. (eds.) *L'art au quotidien. Objets ornés du Paléolithique supérieur. Actes du colloque international L'art au quotidien. Objets ornés du Paléolithique supérieur, les Eyzies-de-Tayac, juin 2014.*, Numéro spécial (Paleo). In press.

47. Kalicki, T., Kozłowski, J. K., Krzemińska, A., Sobczyk, K. & Wojtal, P. The formation of mammoth bone accumulation at the Gravettian site Kraków-Spadzista B+B1. *Folia Quaternaria* **77**, 5–30 (2007).

48. Kozłowski, J. K., Van Vliet, B., Sachse-Kozłowska, E., Kubiak, H. & Zakrzewska, G. Upper Paleolithic site with dwellings of mammoth bones e Cracow, Spadzista street B. *Folia Quaternaria* **44**, 1–110 (1974).

49. Wojtal, P. & Sobczyk, K. Man and woolly mammoth at the Kraków Spadzista street (B) – taphonomy of the site. *J. Archaeol. Sci.* **32**, 193–206 (2005).

50. Wilczyński, J. The Gravettian and Epigravettian lithic assemblages from Kraków-Spadzista B+B 1: dynamic approach to the technology. *Folia Quaternaria* **77**, 37–96 (2007).

51. Wilczyński, J., Wojtal, P. & Sobczyk, K. Spatial organization of the Gravettian mammoth hunters' site at Kraków Spadzista (southern Poland). *J. Archaeol. Sci.* **39**, 3627–3642 (2012).

52. Wilczyński, J., Wojtal, P., Sobieraj, D. & Sobczyk, K. Kraków Spadzista trench C2: New research and interpretations of Gravettian settlement. *Quat. Int.* **359–360**, 96–113 (2015).

53. Kufel-Diakowska, B., Wilczyński, J., Wojtal, P. & Sobczyk, K. Mammoth hunting – impact traces on backed implements from a mammoth bone accumulation at Kraków Spadzista (southern Poland). *J. Archaeol. Sci.* **65**, 122–133 (2016).

54. Lipecki, G. & Wojtal, P. Carnivores from the open-air Gravettian site Kraków Spadzista. In Wojtal, P., Wilczyński, J. & Haynes, G. (eds.) *A Gravettian Site in Southern Poland: Kraków Spadzista*, 117–158 (ISEA PAS, Kraków, 2015).

55. Bocherens, H. *et al.* Paleobiological implications of the isotopic signatures (^{13}C,^{15}N) of fossil mammal collagen in Scladina Cave (Sclayn, Belgium). *Quat. Res.* **48**, 370–380 (1997).

56. Bocherens, H., Drucker, D., Billiou, D. & Moussa, I. Une nouvelle approche pour évaluer l'état de conservation de l'os et du collagène pour les mesures isotopiques (datation au radiocarbone, isotopes stables du carbone et de l'azote). *Anthropologie* **109**, 557–567 (2005).

57. DeNiro, M. J. Postmortem preservation and alteration of in vivo bone collagen isotope ratios in relation to palaeodietary reconstruction. *Nature* **317**, 806–809 (1985).

58. Drucker, D. G. *et al.* Tracking possible decline of woolly mammoth during the Gravettian in Dordogne (France) and the Ach valley (Germany) using multi-isotope tracking (^{13}C, ^{14}C, ^{15}N, ^{34}S, ^{18}O). *Quat. Int.* **359–360**, 304–317 (2015).

59. Talamo, S. *et al.* Direct radiocarbon dating and genetic analyses on the purported neanderthal mandible from the Monti Lessini (Italy). *Sci. Rep.* **6**, 29144 (2016).

60. Welker, F., Soressi, M., Rendu, W., Hublin, J.-J. & Collins, M. J. Using ZooMS to identify fragmentary bone from the late Middle/Early Upper Palaeolithic sequence of Les Cottés, France. *J. Archaeol. Sci.* **54**, 279–286 (2015).

61. Welker, F. *et al.* Palaeoproteomic evidence identifies archaic hominins associated with the Châtelperronian at the Grotte du Renne. *Proc. Natl. Acad. Sci. U. S. A.* **113**, 11162–11167 (2016).

62. Dabney, J. *et al.* Complete mitochondrial genome sequence of a Middle Pleistocene cave bear reconstructed from ultrashort DNA fragments. *Proc. Natl. Acad. Sci. U. S. A.* **110**, 15758–15763 (2013).

63. Meyer, M. & Kircher, M. Illumina sequencing library preparation for highly multiplexed target capture and sequencing. *Cold Spring Harb. Protoc.* **2010**, db.prot5448 (2010).

64. Kircher, M., Sawyer, S. & Meyer, M. Double indexing overcomes inaccuracies in multiplex sequencing on the illumina platform. *Nucleic Acids Res.* **40**, e3 (2012).

65. Schuenemann, V. J. *et al.* Genome-wide comparison of medieval and modern *Mycobacterium leprae*. *Science* **341**, 179–183 (2013).

66. Altschul, S. F., Gish, W., Miller, W., Myers, E. W. & Lipman, D. J. Basic local alignment search tool. *J. Mol. Biol.* **215**, 403–410 (1990).

67. Maricic, T., Whitten, M. & Pääbo, S. Multiplexed DNA sequence capture of mitochondrial genomes using PCR products. *PLoS One* **5**, e14004 (2010).

68. Peltzer, A. *et al.* EAGER: efficient ancient genome reconstruction. *Genome Biol.* **17**, 1–14 (2016).

69. Andrews, S. FastQC: A quality control tool for high throughput sequence data (2010).

70. Li, H. & Durbin, R. Fast and accurate short read alignment with Burrows-Wheeler transform. *Bioinformatics* **25**, 1754–1760 (2009).

71. García-Alcalde, F. *et al.* Qualimap: evaluating next-generation sequencing alignment data. *Bioinformatics* **28**, 2678–2679 (2012).

72. Jónsson, H., Ginolhac, A., Schubert, M., Johnson, P. L. F. & Orlando, L. mapDamage2.0: fast approximate bayesian estimates of ancient DNA damage parameters. *Bioinformatics* **29**, 1682–1684 (2013).

73. McKenna, A. *et al.* The genome analysis toolkit: a MapReduce framework for analyzing next-generation DNA sequencing data. *Genome Res.* **20**, 1297–1303 (2010).

74. DePristo, M. A. *et al.* A framework for variation discovery and genotyping using next-generation DNA sequencing data. *Nat. Genet.* **43**, 491–498 (2011).

75. Van der Auwera, G. A. *et al.* From FastQ data to High-Confidence variant calls: The genome analysis toolkit best practices pipeline. In *Current Protocols in Bioinformatics* (John Wiley & Sons, Inc., 2002).

76. Briggs, A. W. *et al.* Patterns of damage in genomic DNA sequences from a neandertal. *Proc. Natl. Acad. Sci. U. S. A.* **104**, 14616–14621 (2007).

77. Chang, D. *et al.* The evolutionary and phylogeographic history of woolly mammoths: a comprehensive mitogenomic analysis. *Sci. Rep.* **7**, 44585 (2017).

78. Kearse, M. *et al.* Geneious basic: an integrated and extendable desktop software platform for the organization and analysis of sequence data. *Bioinformatics* **28**, 1647–1649 (2012).

79. Keane, T. M., Creevey, C. J., Pentony, M. M., Naughton, T. J. & Mclnerney, J. O. Assessment of methods for amino acid matrix selection and their use on empirical data shows that ad hoc assumptions for choice of matrix are not justified. *BMC Evol. Biol.* **6**, 29 (2006).

80. Nguyen, L.-T., Schmidt, H. A., von Haeseler, A. & Minh, B. Q. IQ-TREE: a fast and effective stochastic algorithm for estimating maximum-likelihood phylogenies. *Mol. Biol. Evol.* **32**, 268–274 (2015).

81. Darriba, D., Taboada, G. L., Doallo, R. & Posada, D. jModelTest 2: more models, new heuristics and parallel computing. *Nat. Methods* **9**, 772 (2012).

82. Tamura, K. & Nei, M. Estimation of the number of nucleotide substitutions in the control region of mitochondrial DNA in humans and chimpanzees. *Mol. Biol. Evol.* **10**, 512–526 (1993).

83. Gilbert, M. T. P. *et al.* Intraspecific phylogenetic analysis of Siberian woolly mammoths using complete mitochondrial genomes. *Proc. Natl. Acad. Sci. U. S. A.* **105**, 8327–8332 (2008).

84. Palkopoulou, E. *et al.* Holarctic genetic structure and range dynamics in the woolly mammoth. *Proceedings of the Royal Society B: Biological Sciences* **280**, 20131910 (2013).

85. Jia, F., Lo, N. & Ho, S. Y. W. The impact of modelling rate heterogeneity among sites on phylogenetic estimates of intraspecific evolutionary rates and timescales. *PLoS One* **9**, e95722 (2014).

86. Tamura, K., Stecher, G., Peterson, D., Filipski, A. & Kumar, S. MEGA6: Molecular evolutionary genetics analysis version 6.0. *Mol. Biol. Evol.* **30**, 2725–2729 (2013).

87. Ronquist, F. *et al.* MrBayes 3.2: Efficient bayesian phylogenetic inference and model choice across a large model space. *Syst. Biol.* **61**, 539–542 (2012).

88. Enk, J. *et al.* *Mammuthus* population dynamics in Late Pleistocene North America: Divergence, phylogeography, and introgression. *Front. Ecol. Evol.* **4** (2016).

89. Duchêne, S., Archer, F. I., Vilstrup, J., Caballero, S. & Morin, P. A. Mitogenome phylogenetics: the impact of using single regions and partitioning schemes on topology, substitution rate and divergence time estimation. *PLoS One* **6**, e27138 (2011).

90. R Core Team. *R: A language and environment for statistical computing* (R Foundation for Statistical Computing, Vienna, Austria, 2015).

91. Paradis, E., Claude, J. & Strimmer, K. APE: Analyses of phylogenetics and evolution in R language. *Bioinformatics* **20**, 289–290 (2004).

92. Cingolani, P. *et al.* A program for annotating and predicting the effects of single nucleotide polymorphisms, SnpEff: SNPs in the genome of *Drosophila melanogaster* strain w1118; iso-2; iso-3. *Fly* **6**, 80–92 (2012).

93. Münzel, S. C., Wolf, S., Drucker, D. G. & Conard, N. J. The exploitation of mammoth in the Swabian Jura (SW-Germany) during the aurignacian and gravettian period. *Quat. Int.* **445**, 184–199 (2017).

Supplementary Figures

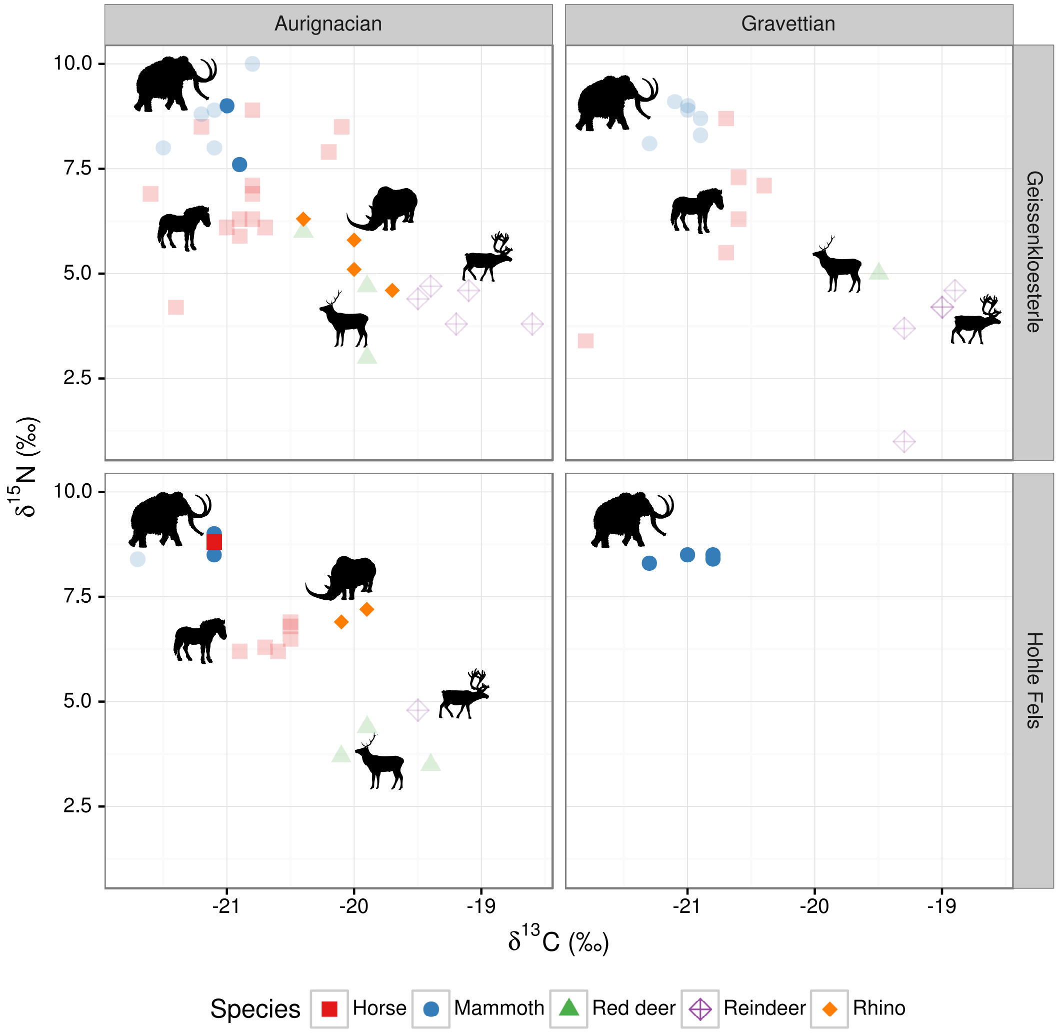

Figure S1. Isotopic values of herbivores from Geißenklösterle (GK) and Hohle Fels (HF) between the Aurignacian and Gravettian. Transparent symbols indicate previously published data[58, 93], bright symbols indicate new values presented here. Asterixis (*) indicate samples deriving from a transitional Aurignacian-Gravettian archaeological horizon (IIe). Geißenklösterle shows the horses spreading into the normally distinct isotopic position of the woolly mammoth in both the Aurignacian and Gravettian, whereas at Hohle Fels the woolly mammoth maintains the distinct position, albeit with a low sample number. The single horse at Hohle Fels reaching δ^{15}N values similar to woolly mammoth derives from a transitional horizon. Mammoth and Horse silhouettes by Dorothée Drucker. Remaining species are in the public domain and taken from phylopic.org

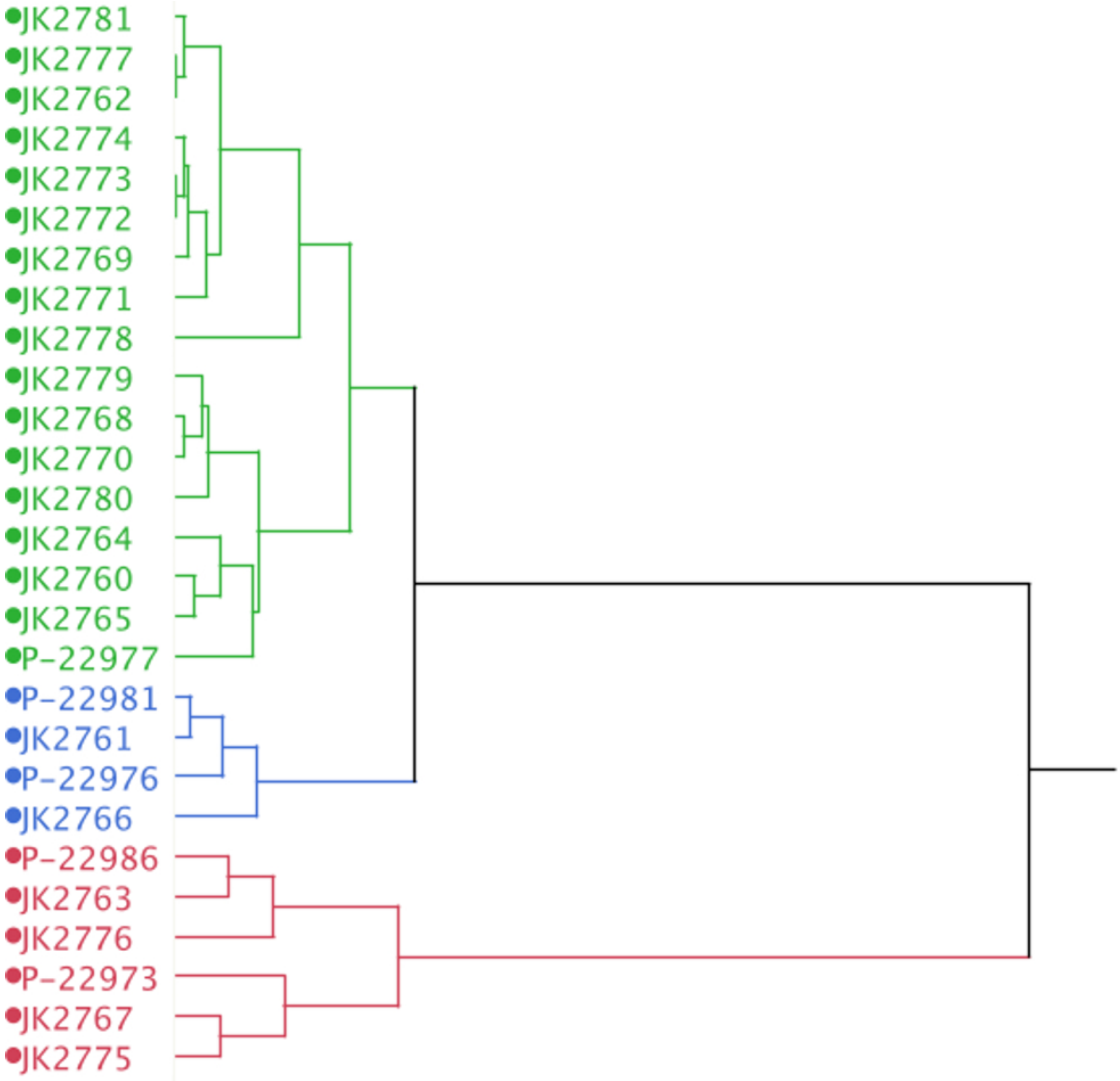

Figure S2. Cluster analysis using Ward's minimum variance of ^{13}C and ^{15}N isotopic values of samples from Geißenklösterle and Hohle Fels analysed in this study. Green and blue branches clusters consist of mammoth individuals whereas red indicates a rhino cluster. Image generated by JMP.

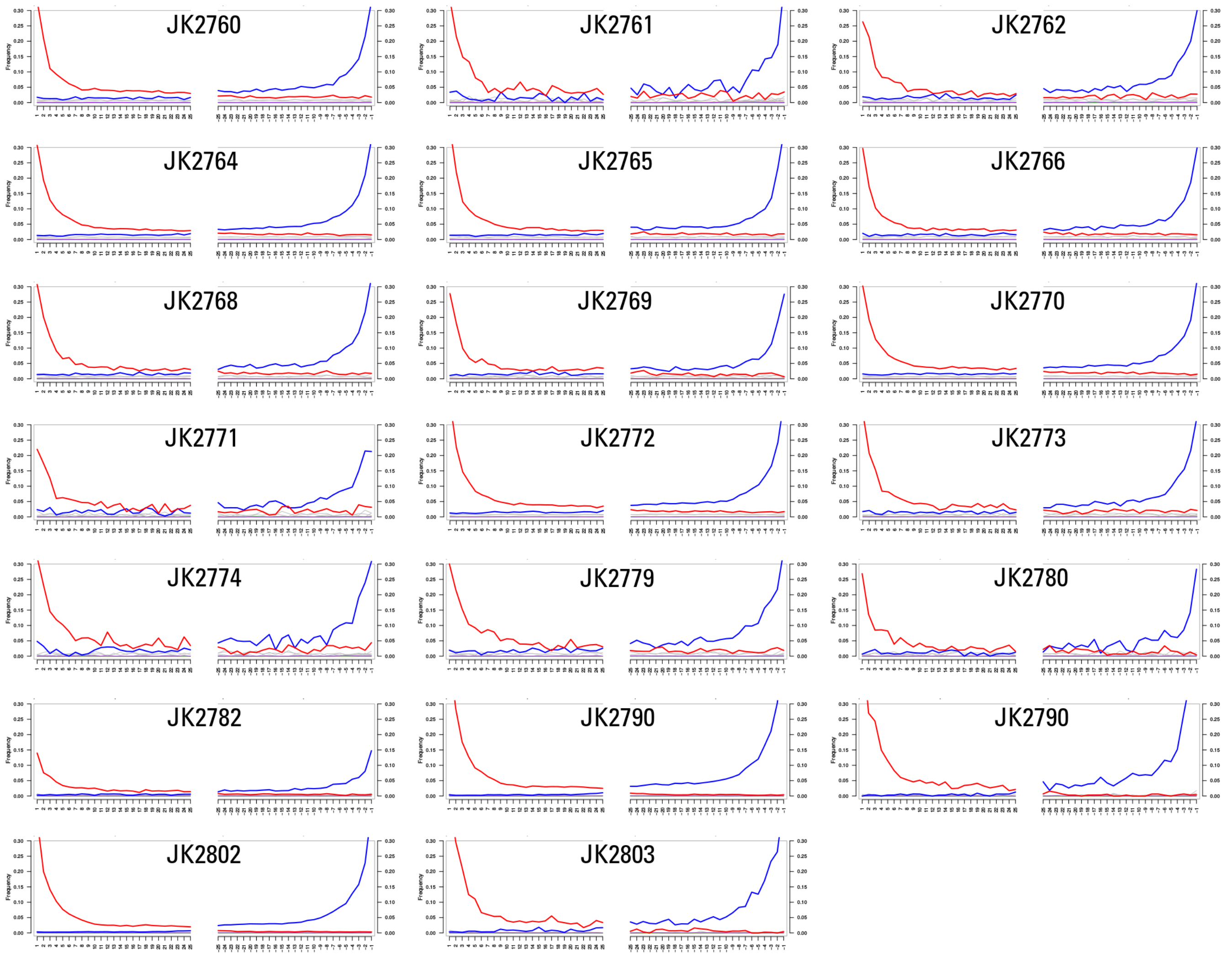

Figure S3. Damage patterns of all captured 20 woolly mammoth mitochondrial genomes with greater than x3 coverage and 66% complete, as generated by mapDamage v2[72]. The younger Kesslerloch sample (JK2782, 15 ka cal BP) has a lower frequency of C to T deamination substitutions at around 15% compared to >25% of the older Kraków Spadzista (JK2790 - JK2803, 26 ka cal BP) and Geißenklösterle and Hohle Fels (JK2760 - JK2780, >31 ka cal BP).

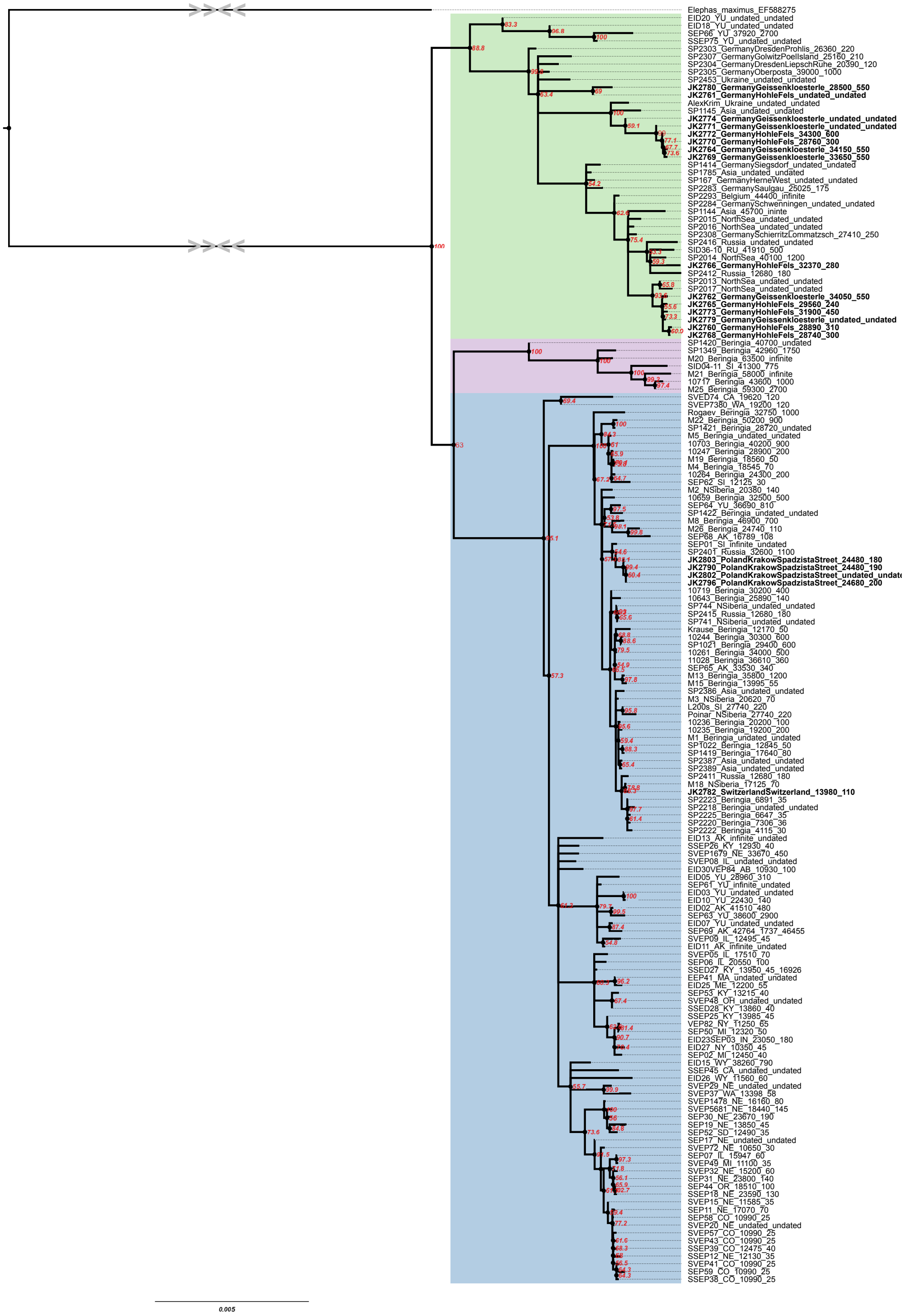

Figure S4. Neighbour joining tree based on all mitochondrial genomes included in Chang et al.[77] and those generated in this study (in bold). The tree was generated in Geneious vR8 with 1000 bootstraps and the TN model. Statistical support of a node is indicated in red as a percentage of 1000 bootstrapped trees. Grey arrows represent an artificial truncation of branches leading to the outgroup due to space restrictions. Clades as previously assigned in Palkopoulou et al. are represented as follows: Blue - Clade I; Purple - Clade II; Green - Clade III. Tree was generated using Figtree (tree.bio.ed.ac.uk/software/figtree/) and modified in Inkscape (inkscape.org).

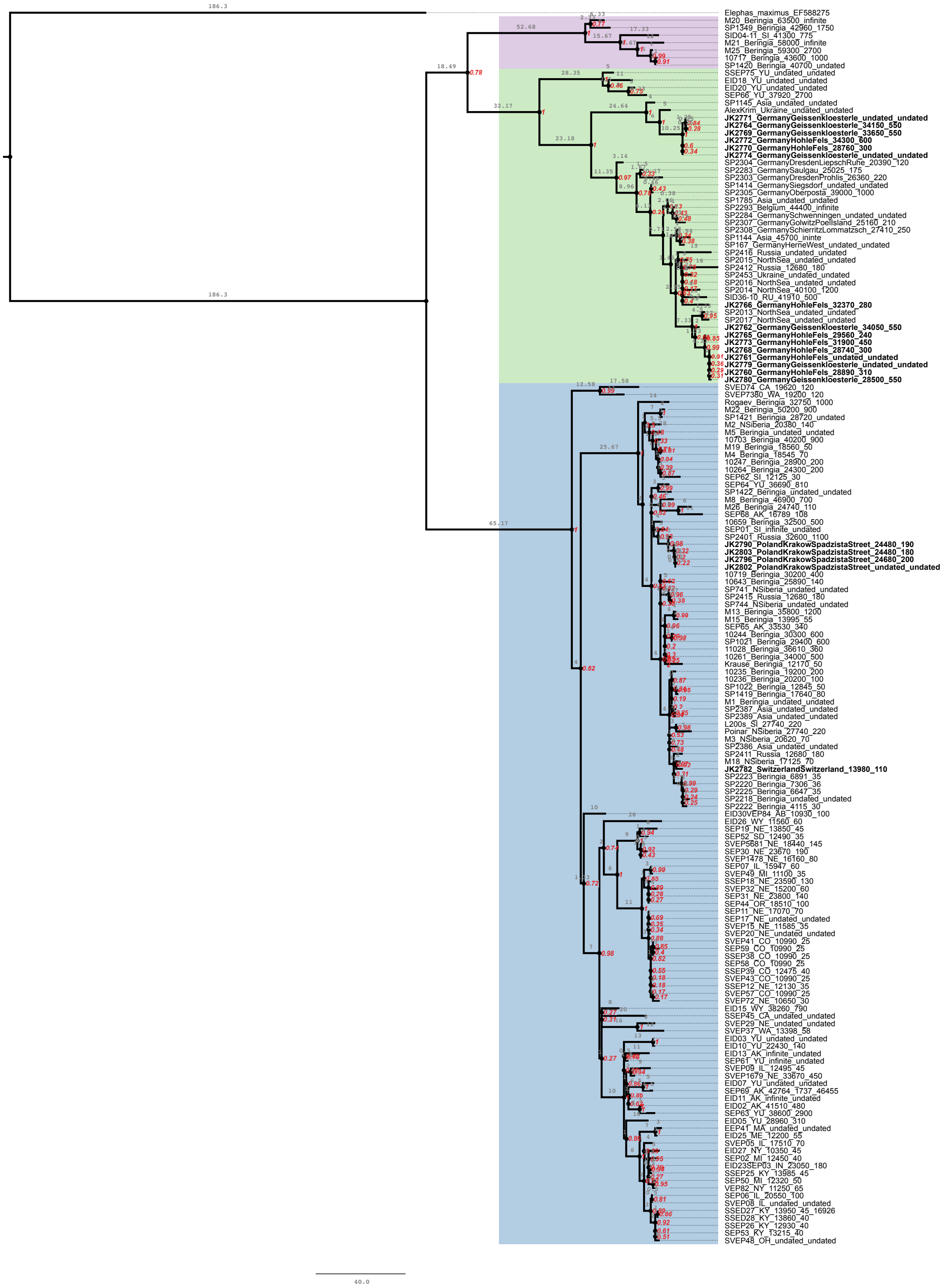

Figure S5. Maximum Parsimony tree based on all mitochondrial genomes included in Chang et al.[77] and those generated in this study (in bold). The tree was generated in MEGA v6[86] with 1000 bootstraps and the Subtree-Pruning-Regrafting search method. Statistical support of a node is indicated in red as a percentage of 1000 bootstrapped trees. Branch lengths in the form of number of mutations are indicated in grey. Clades as previously assigned in Palkopoulou et al. are represented as follows: Blue - Clade I; Purple - Clade II; Green - Clade III. Tree was generated using Figtree (tree.bio.ed.ac.uk/software/figtree/) and modified in Inkscape (inkscape.org).

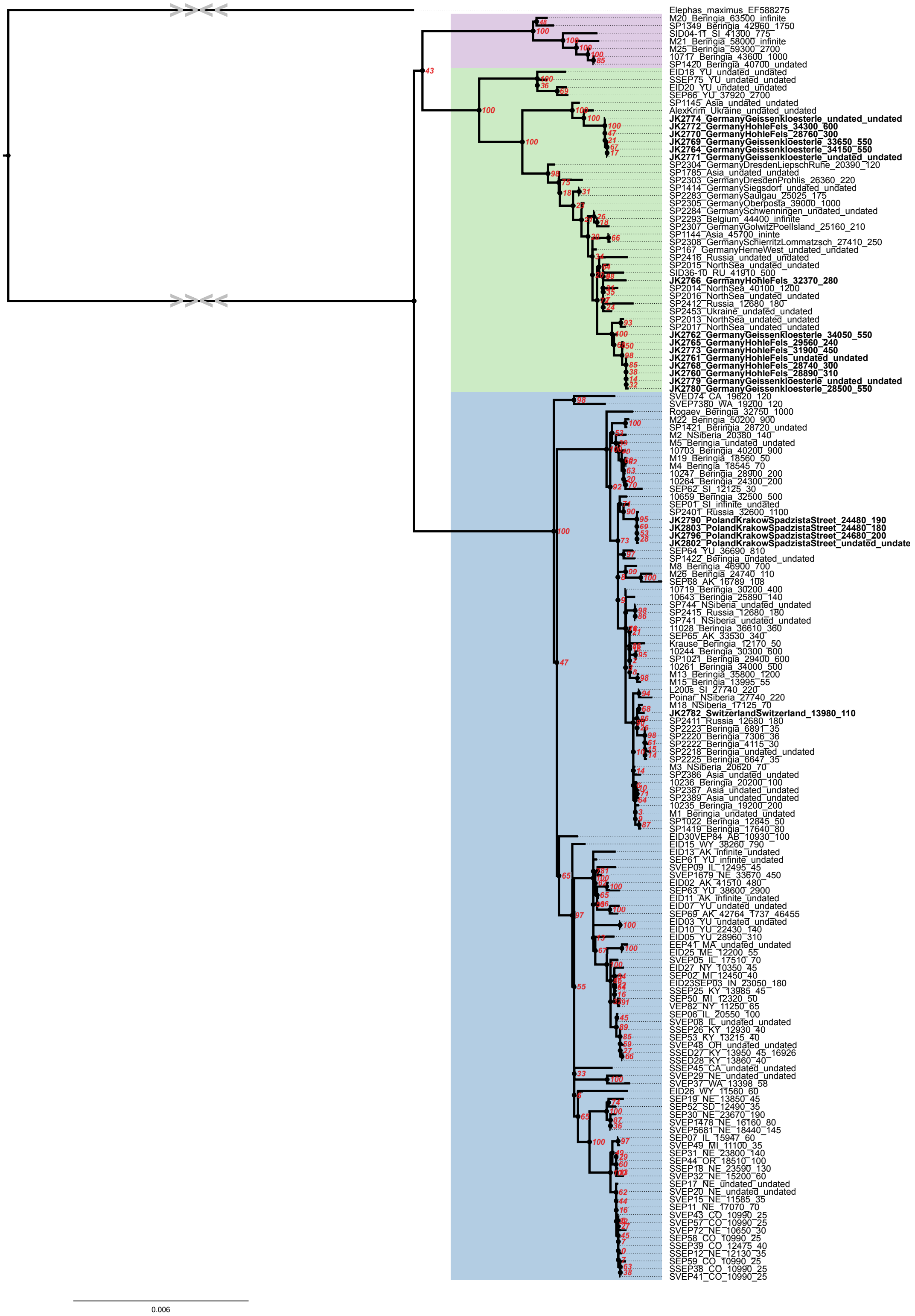

Figure S6. Maximum Likelihood tree based on all mitochondrial genomes included in Chang et al.[77] and those generated in this study (in bold). The tree was generated using IQ-Tree 1.4.2[80] to generate a Maximum Likelihood tree, with 1000 bootstraps and TN+G4 model. Statistical support of a node is indicated in red as a percentage of 1000 bootstrapped trees. Grey arrows represent an artificial truncation of branches leading to the outgroup due to space restrictions. Clades as previously assigned in Palkopoulou et al. are represented as follows: Blue - Clade I; Purple - Clade II; Green - Clade III. Tree was generated using Figtree (tree.bio.ed.ac.uk/software/figtree/) and modified in Inkscape (inkscape.org).

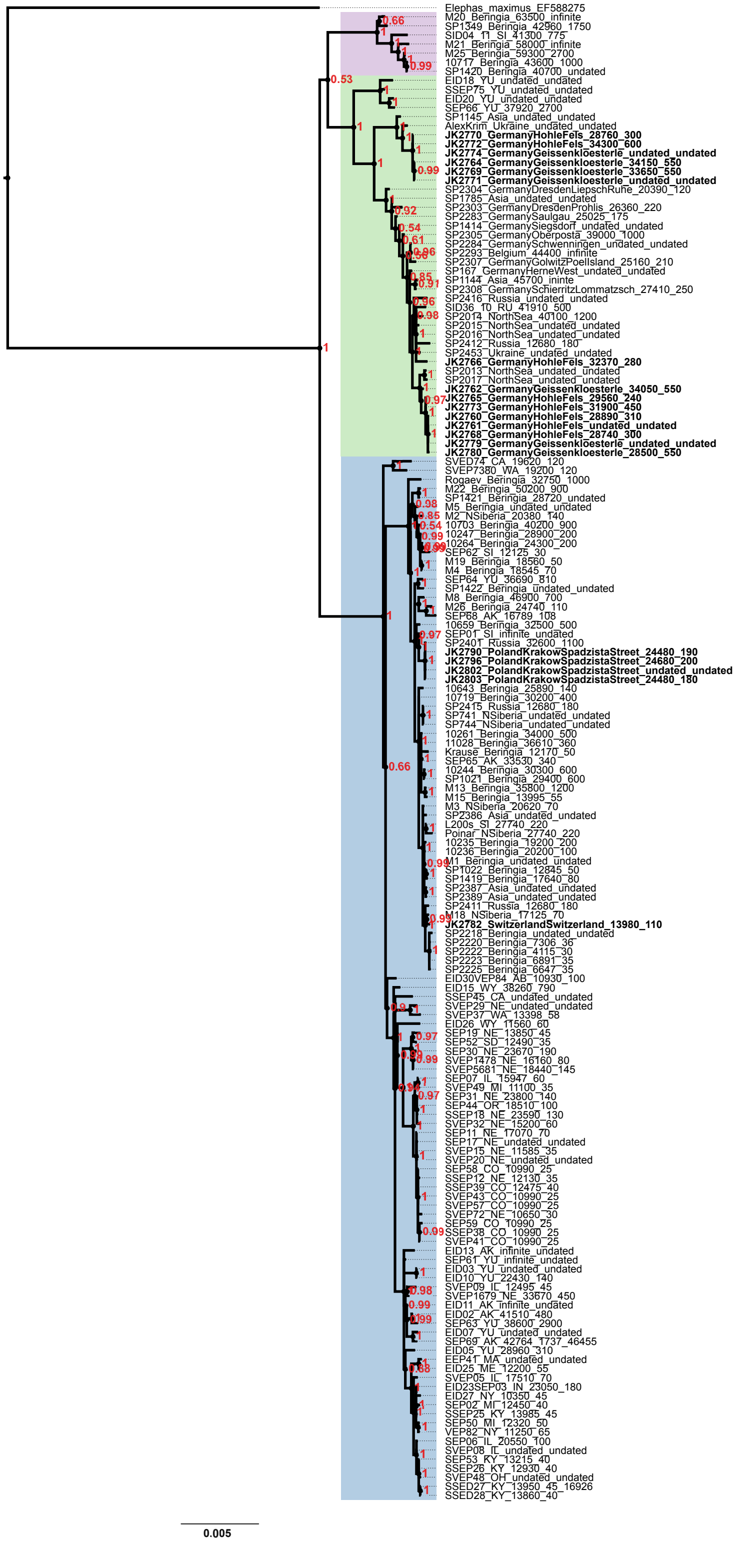

Figure S7. Bayesian tree based on all mitochondrial genomes included in Chang et al.[77] and those generated in this study (in bold). This was generated with MrBayes v3.2.6[87], with approximation of the TN+G model. The analysis was run for 100,000,000 steps sampling every 10,000. Statistical support of a node in the form of posterior probability is indicated in red. Grey arrows represent an artificial truncation of branches leading to the outgroup due to space restrictions. Clades as previously assigned in Palkopoulou et al. are represented as follows: Blue - Clade I; Purple - Clade II; Green - Clade III. Tree was generated using Figtree (tree.bio.ed.ac.uk/software/figtree/) and modified in Inkscape (inkscape.org).

Ancestral	Clade I	Clade II	Clade III	How Processed
G	A	G	A	(Clade II)(Clade I, Clade III)
A	*A*	*A*	*A*	*No SNP - ignored*
T	C	C	T	(Clade III)(Clade I, Clade II)
T	*C*	*C*	*G*	*Independent mutations - ignored*
A	A	T	T	(Clade I)(Clade II, Clade III)
C	T	C	T	(Clade II)(Clade I, Clade III)
A	G	A	G	(Clade II)(Clade I, Clade III)
C	T	T	C	(Clade III)(Clade I, Clade II)
A	*C*	*C*	*G*	*Independent mutations - ignored*
T	*T*	*T*	*T*	*No SNP - ignored*

Grouping	Count
(Clade I)(Clade II, Clade III)	1
(Clade II)(Clade I, Clade III)	3
(Clade III)(Clade I, Clade II)	2

Figure S8. Schematic of the concept behind assessment of 'shared' derived SNPs. In an alignment (left) of three individuals from each clade, when two clades have a derived SNPs but the other individual maintains the ancestral state, the grouping is counted (right). All other positions are ignored. The grouping counts on the right are then averaged across the three different sets of individuals.
